# Influenza A virus infection impairs neuronal activity in human iPSC-derived NGN2 neural co-cultures

**DOI:** 10.1186/s40478-026-02292-0

**Published:** 2026-04-18

**Authors:** Feline F. W. Benavides, Annabel L. V. Kempff, Hilde Smeenk, Bas Lendemeijer, Marla Lavrijsen, Johan A. Slotman, Steven A. Kushner, Femke M. S. de Vrij, Lisa Bauer, Debby van Riel

**Affiliations:** 1 Department of Viroscience, Erasmus MC, Rotterdam, The Netherlands; 2 Department of Psychiatry, Erasmus MC, Rotterdam, The Netherlands; 3 Optical Imaging Centre and Department of Pathology, Erasmus MC, Rotterdam, The Netherlands; 4 Stavros Niarchos Foundation (SNF) Center for Precision Psychiatry & Mental Health, Columbia University, New York, NY USA; 5 Department of Psychiatry, Columbia University Irving Medical Center, New York, NY USA

**Keywords:** Influenza A viruses, hiPSC-derived neural co-culture, NGN2 neuron, Micro-electrode array system (MEA), Neuronal activity, Cognitive impairment

## Abstract

Influenza A virus (IAV) infection is associated with a wide variety of neurological complications, of which mild complications like impaired cognitive functioning are most prominent. Even though several studies have shown that many influenza viruses can enter the CNS, the neuropathogenesis of seasonal (H3N2 and H1N1) and pandemic (pH1N1 2009) IAV infections is poorly understood. Therefore, we aimed to investigate the cellular tropism, replication efficiency and associated functional consequences using a human stem cell-derived neural co-culture model of neurons and astrocytes. All viruses were able to infect neurons in the co-culture model, although this infection did not result in efficient replication and release of progeny virus. In addition, infection did not result in visible cell death or apoptosis. However, functional analyses revealed that IAV inoculation resulted in a reduction of spontaneous neural activity and a partial reduction of neural excitability. This study shows that seasonal and pandemic IAVs can disrupt neural homeostasis, without efficient virus replication or the induction of cell death. These functional changes in neural activity can contribute to cognitive problems during IAV infections in the acute and potentially post-acute phase of the infection.

**Supplementary Information:**

The online version contains supplementary material available at 10.1186/s40478-026-02292-0.

## Introduction

Influenza A virus infections cause morbidity and mortality worldwide. Even though it is considered a respiratory pathogen, neurological symptoms can occur [1–3]. Neurological complications vary in symptoms and onset, and differ among influenza A virus subtypes. Zoonotic viruses, like H5Nx viruses, are particularly well known for their ability to cause severe neurological complications in a wide range of mammals, including humans [4]. In contrast, seasonal and pandemic influenza A viruses generally cause milder neurological complications, such as cognitive problems, and rarely more severe complications [5, 6].

There is evidence from patients and in vivo studies that seasonal and pandemic influenza A viruses can enter the CNS. In humans, both viral RNA and antigen have been detected in neurons and glial cells in the brain post-mortem, and occasionally in cerebrospinal fluid [3, 7–13]. In ferrets, infectious virus or viral RNA has been detected in the olfactory bulb or brains of seasonal H3N2 virus inoculated ferrets [5, 14–20] and in the olfactory bulb, cerebrum and cerebellum of pandemic H1N1 2009 (pH1N1 2009) virus inoculated ferrets [5, 21]. In pandemic 1918 H1N1 inoculated ferrets, infectious virus, viral RNA and viral antigen were detected in neurons in the olfactory bulb, cerebrum, brain stem and trigeminal nerve [22]. In mouse studies, neuroinvasion has been observed with mouse-adapted H1N1 viruses (H1N1 WSN) and H3N2 virus [23, 24], while the pandemic H1N1 2009 virus (pH1N1 2009) and mouse-adapted H1N1 (H1N1 PR8) could not be detected in the brain [23, 25, 26]. Even though neuroinvasion is observed for a variety of seasonal and pandemic influenza viruses, the neurotropism has not been studied extensively. Previous in vitro studies have shown that pH1N1 2009, seasonal H3N2, H1N1 WSN and H1N1 PR8 viruses can infect and replicate in neural cells [24, 27–29]. However, these studies use either cell lines, lab-adapted or mouse-adapted viruses and have not been performed in more relevant human neural culture systems in combination with relevant clinical isolates.

Knowledge of the neurovirulent potential of seasonal and pandemic influenza A viruses is limited. For seasonal viruses, mild neurological complications, like learning and memory problems are most prominent [30], but severe neurological complications like encephalitis, transverse myelitis, brain edema or loss of vision have been reported [31–38]. For pandemic viruses, like during the 2009 H1N1 pandemic, severe neurological symptoms were more frequently reported and included seizures and encephalopathies [39–41], but also milder symptoms like confusion, dizziness and headache [2, 38, 41–43]. Additionally, a recent study revealed that (severe) influenza A virus infections increase the risk for neurodegenerative diseases [44]. Even though neurological complications associated with influenza A virus infections have been described since 1918 [45], comprehensive knowledge about the neuropathogenesis, and especially that of less severe complications, such as memory and learning problems, is still lacking.

Neurodegenerative and neuropsychiatric diseases, especially those with learning and memory problems, are associated with neuronal cell death, decreased neural activity, and impaired synaptic plasticity [46, 47]. Influenza A virus infection could lead to cell death by the cytopathic effect of the infection or through the direct or indirect induction of apoptosis. An increase of the apoptotic marker cleaved caspase-3 was detected in neurons and glial cells in the post-mortem brains of influenza patients [48] and in vitro in neurons, astrocytes, and neural stem cells after inoculation with influenza A virus [29, 49, 50]. Neural activity, which refers to the communication between neural cells, is known to affect learning and memory formation, but this has not been studied in the context of influenza A viruses. Synaptic plasticity, which is the ability to form and strengthen connections between neuronal cells after stimulation, can be studied by investigating neuronal excitability by employing long-term potentiation (LTP) protocols. LTP was reduced in organotypic hippocampal slices of mice inoculated with a mouse-adapted pandemic H3N2 virus in a time-dependent manner [23], which was associated with a decrease in dendritic spine density [23]. In addition, in mouse-adapted pandemic H1N1 virus inoculated neonatal mice there was a reduction in spontaneous neurotransmission and neuronal excitability [51]. The underlying mechanisms of the changes in neural activity are unclear but might be related to a disbalance in glutamatergic synapse transmission [25].

The aim of this study was to gain insight into the neurotropic and neurovirulent potential of recent seasonal and pandemic influenza A viruses. Therefore, we studied the cell tropism, replication kinetics, immune responses and ability to induce apoptosis of pH1N1 2009 virus, and seasonal H1N1 and H3N2 viruses in a human induced pluripotent stem cell (hiPSC)-derived neural co-culture model that contains neurons and astrocytes. In addition, neural activity and excitability of the neural co-cultures upon virus inoculation were assessed with a micro-electrode array (MEA) platform in order to better understand the underlying mechanisms of neurological complications associated with influenza A virus infection.

## Results

### Inefficient replication of pH1N1 2009, H1N1 2019 and H3N2 2019 viruses in neural co-cultures, consisting of NGN2 neurons and astrocytes

**Fig. 1:**
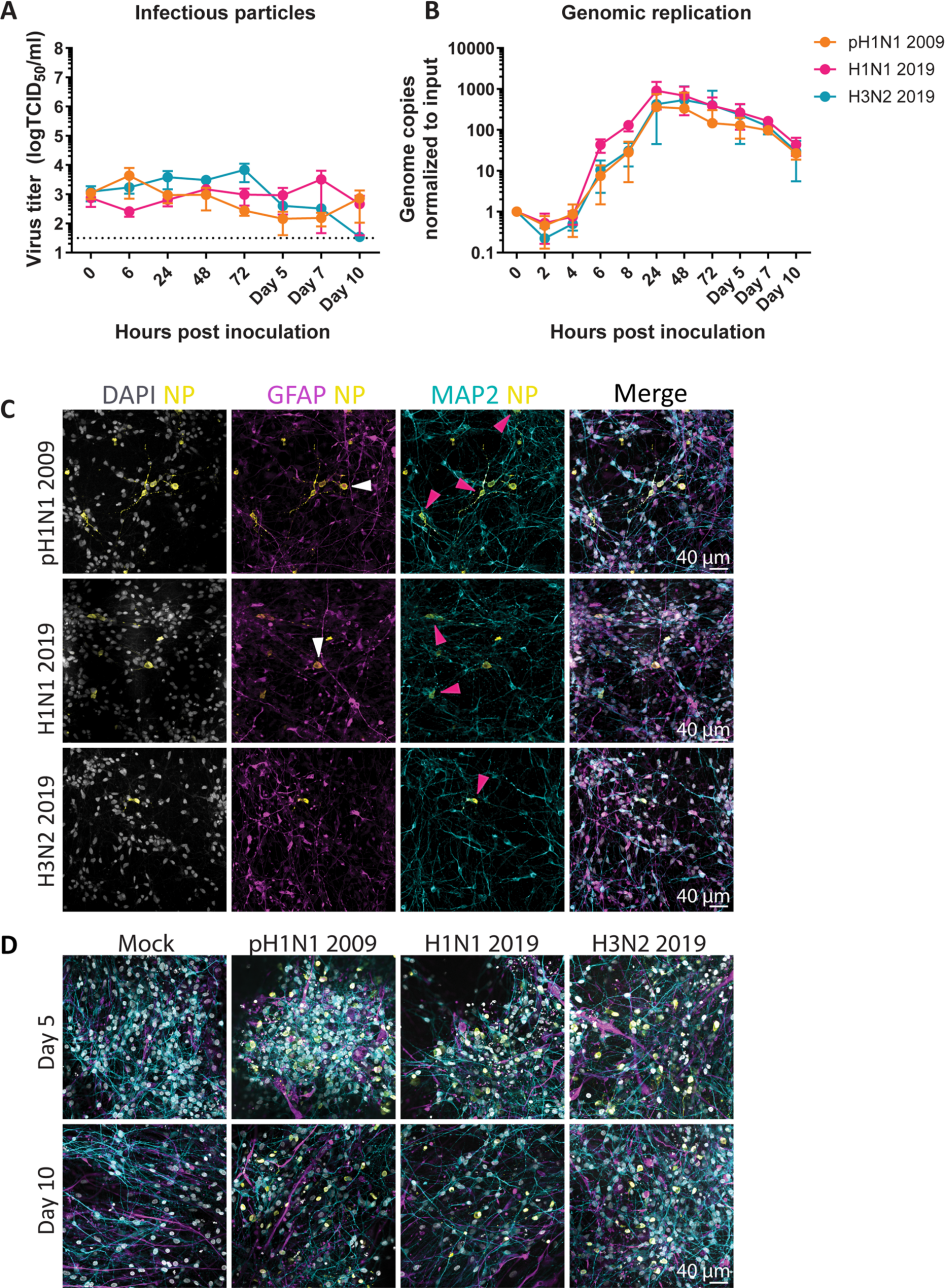
Inefficient replication of pH1N1 2009, H1N1 2019 and H3N2 2019 viruses in neural co-cultures, consisting of NGN2 neurons and astrocytes. Human induced pluripotent stem cell-derived neural co-cultures consisting of NGN2 neurons and astrocytes were inoculated with pH1N1 2009, H1N1 2019 or H3N2 2019 virus with an MOI of 1 at DIV 21. **A** Growth kinetics of pH1N1 2009, H1N1 2019, H3N2 2019 virus. Data represent mean ± standard deviation (SD) and are derived from at least three independent experiments, with three biological replicates and three technical replicates. Dotted line represents lower limited of detection. **B** Intracellular replication kinetics of pH1N1 2009, H1N1 2019 and H3N2 2019 virus. Data represent mean ± SD, and are derived from at least three independent experiments, with one biological replicate and two technical duplicates. **C** Neural co-cultures were fixed 72 h post inoculation and were stained with microtubule-associated protein (MAP2; cyan) as a marker for neurons, glial fibrillary acidic protein (GFAP; magenta) as a marker for astrocytes, and influenza A virus nucleoprotein (NP; yellow) was used to identify infected cells. Cells were counterstained with Hoechst (grey) to visualize the nuclei. Data shown are representative examples from three independent experiments. Co-localization between NP and GFAP is indicated with white arrow, and co-localization between NP and MAP2 with a pink arrow. **D** Neural co-cultures were fixed 5- and 10-days post inoculation and stained for MAP2, GFAP, NP and DAPI. Data shown are representative examples from three independent experiments

To evaluate the replication efficiency of pH1N1 2009, H3N2 2019 and H1N1 2019 viruses, hiPSC-derived neural co-cultures, consisting of Neurogenin-2 (NGN2) neurons and astrocytes, were inoculated with an multiplicity of infection (MOI) of 1. No increase in infectious virus was observed with any of the viruses up to 10 days post inoculation (dpi; Fig. 1A), despite efficient replication in Madin-Darby Canine Kidney (MDCK) cells (Figure S1A). It is possible that inefficient replication occurred in the neural co-cultures inoculated with pH1N1 2009, H1N1 2019 or H3N2 2019 virus as virus titers remained stable and detectable over time, whereas virus titers in culture medium decreased to undetectable levels at 10 dpi (Figure S1B). In addition, intracellular viral RNA levels increased for all three viruses and peaked at 24 h post inoculation (hpi; Fig. 1B).

To investigate the neurotropism, neural co-cultures were fixed at 72 hpi and stained for virus nucleoprotein (NP), microtubule-associated protein (MAP2, neuronal marker) and glial fibrillary acidic protein (GFAP, astrocytic marker). With all viruses, NP^+^ cells were observed in neural co-cultures (Fig. 1C). Co-localization of NP was visually predominantly observed with MAP2 (Fig. 1C, pink arrows), although NP occasionally co-localized with GFAP in pH1N1 2009 and H1N1 2019 virus inoculated cultures (Fig. 1C, white arrows). Pixel-based quantification showed NP^+^ signal co-localized with both MAP2^+^ and GFAP^+^ signal at day 3 for pH1N1 2009 and H1N1 2019, and less for H3N2 2019 (Figure S1CD). In neural co-cultures fixed at 5- and 10 dpi, similar amounts of NP^+^ cells were detected for all three viruses compared to 3 dpi (Fig. 1D). This was verified with pixel-based quantification (Figure S1CD).

### No induction of the apoptotic marker cleaved caspase-3 and anti-viral or innate immune responses in neural co-cultures inoculated with pH1N1 2009, H1N1 2019 or H3N2 2019 virus

**Fig. 2:**
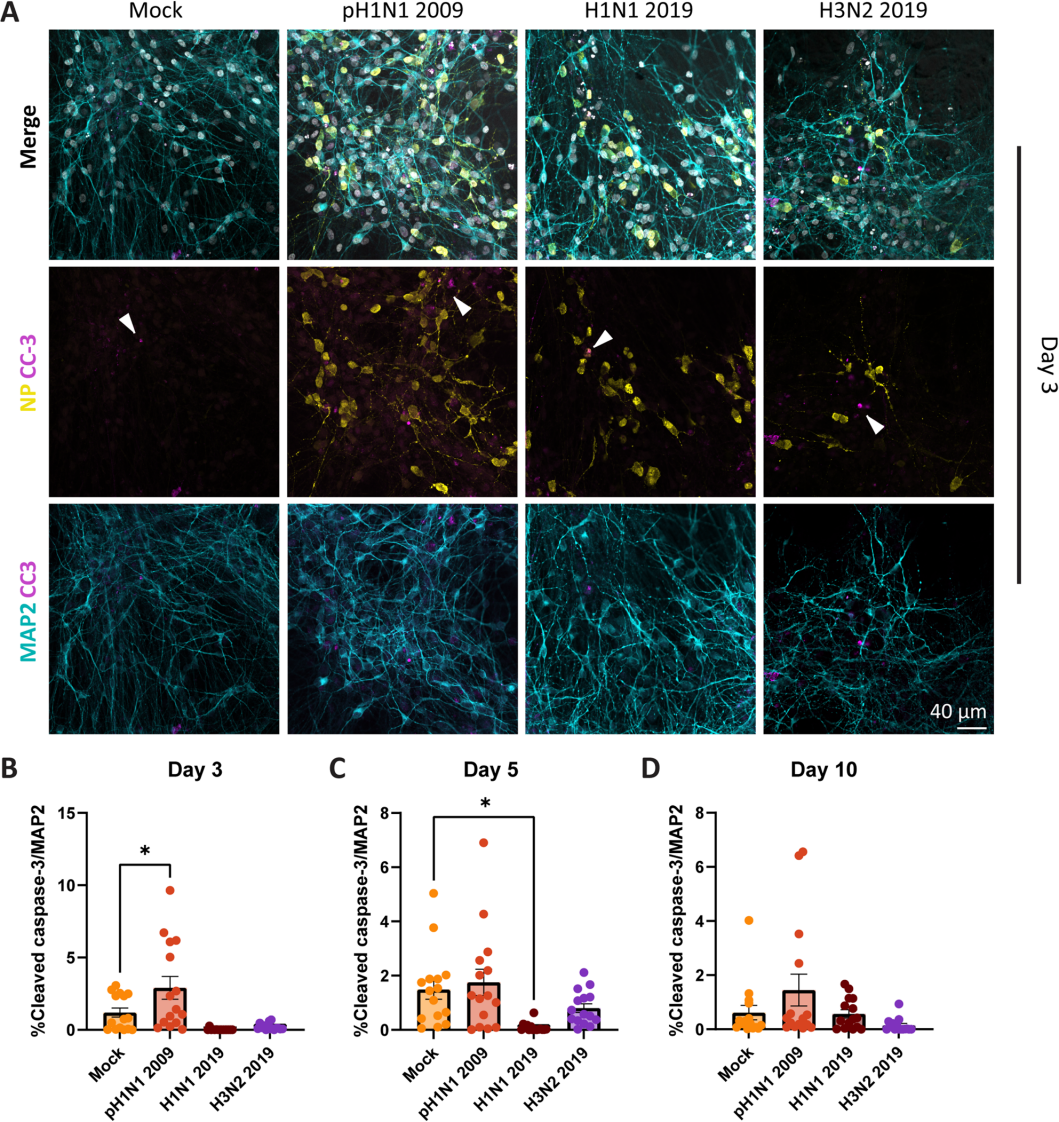
No induction of the apoptotic marker cleaved caspase-3 in the neural co-cultures inoculated with pH1N1 2009, H1N1 2019 or H3N2 2019 virus. **A** Neural co-cultures were fixed 3 days post inoculation (dpi) and were stained with microtubule-associated protein (MAP2; cyan) as a marker for neurons, cleaved caspase-3 (CC-3; magenta, indicated with white arrows) as a marker for apoptosis, and influenza A virus nucleoprotein (NP; yellow) to identify infected cells. Cells were counterstained with Hoechst (grey) to visualize the nuclei. Data shown are representative examples from three independent experiments. Pixel-based quantification was performed to detect the cleaved-caspase 3^+^ signal over MAP2^+^ signal at 3 (**B**), 5 (**C**) and 10 (**D**) dpi. Data represent mean ± SEM, and are derived from three independent experiments, with five technical replicates per experiment. Statistics were tested with an one-way ANOVA test, and corrected for multiple hypothesis testing using the Benjamini–Hochberg method (*q < Q with Q < 0.05, individual *p*-values in Table S1). Asterisks indicate a positive discovery (*)

There was no morphological evidence for cell death in the virus inoculated neural co-cultures by visual inspection through light microscope, like cell debris, curling of neural co-cultures or virus-induced cytopathic effects. However, to exclude direct or indirect virus-induced apoptosis in the neural co-cultures, we studied the expression of cleaved caspase-3. Neural co-cultures were fixed at 3-, 5- and 10 dpi and stained for the apoptotic marker cleaved caspase-3 in combination with NP. Cleaved caspase-3 was detected in a few cells in all neural co-cultures, including mock, on day 3, 5 and 10 (Figs. 2A and S2AB, respectively; indicated with white arrows). This baseline level of cleaved caspase-3 was also detected with pixel-based quantification on day 3, 5 and 10 (Fig. 2BCD). Visually, there was no clear difference in the number of cleaved caspase-3 expressed cells between mock and virus inoculated neural co-cultures or over time (Figs. 2A, S2AB). Pixel-based quantification showed a significant increase of cleaved caspase-3 expression in pH1N1 2009 inoculated neural co-cultures compared to mock on day 3 (Fig. 2B; one-way ANOVA (3, 56) = 9.652, *p* < 0.0001, individual *p*-values in Table S1). Also, a significant decrease in the total cleaved caspase-3 expression was observed between H1N1 2019 inoculated and mock neural co-cultures at 5 dpi (Fig. 2C; one-way ANOVA (3, 56) = 5.681, *p* = 0.0018, individual *p*-values in Table S1). There was no significant difference between mock or inoculated groups at 10 dpi (Fig. 1D; one-way ANOVA (3, 56) = 2.697, *p* = 0.0544, individual p-values in Table S1). Additionally, we did not observe co-localization of cleaved caspase-3 and NP visually, suggesting that infection did not directly induce apoptosis.

To evaluate the immune response in the neural co-cultures, cultures were inoculated with pH1N1 2009, H1N1 2019 or H3N2 2019 viruses (MOI 1), mock l or treated with poly I:C to mimic viral infection. At 24- and 72- hours post inoculation, multiple cytokines, such as type I, II and III interferons, were measured and compared to mock neural co-cultures. Type I interferons were not expressed in any of the conditions (Fig. S3A, two-way ANOVA F (4, 33) = 0.9789, *p* = 0.4324; Figure S3B, two-way ANOVA F (4, 33) = 1.377, *p* = 0.2630, respectively, individual *p*-values in Table S2). Type II interferon and in type III interferon λ1 were significantly increased in poly I:C treated cultures only compared to mock cultures at 24 and 72 hpi (Fig. S3C, two-way ANOVA F (4, 17) = 15.25, *p* < 0.0001; Fig. S3D, two-way ANOVA F (4, 33) = 6.287, *p* = 0.0007, respectively, individual p-values in Table S2), while interferon λ2/3 did not differ between groups (Fig. S3E). IL-1β and IL-6 were not different after virus inoculation or treatment with poly I:C (Fig. S3F, two-way ANOVA F (4, 33) = 1.129, *p* = 0.3599; Fig. S3G, two-way ANOVA F (4, 17) = 1.139, *p* = 0.3715, respectively, individual p-values in Table S2). IL-8 was significantly increased in poly I:C treated cultures compared to mock at 24- and 72 hpi (Fig. S3H, two-way ANOVA F (3, 17) = 9.313, *p* = 0.0004, individual p-values in Table S2). IL-10, IL-12p70, IP-10, GM-CSF were not detected (Figs. S3I and S3J, two-way ANOVA F (4, 33) = 1.119, *p* = 0.3644; Fig. S3K; S3L, respectively). Lastly, TNF-α was significantly increased in poly I:C treated cultures compared to mock at 24- and 72 hpi (Figure S3M, two-way ANOVA F (4, 17) = 10.38, *p* = 0.0002, individual *p*-values in Table S2).

### Reduction in spontaneous activity and network activity in neural co-cultures upon inoculation with pH1N1 2009, H1N1 2019 or H3N2 2019 virus

**Fig. 3:**
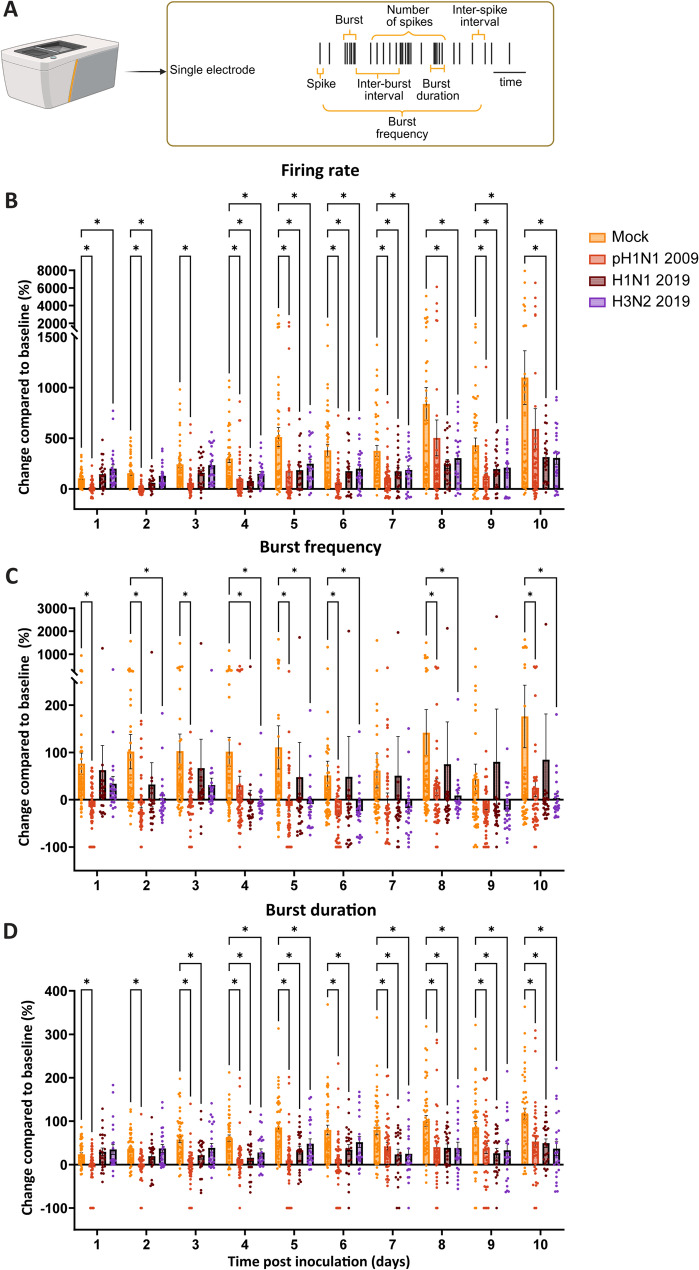
Single electrode spontaneous activity of neural co-cultures inoculated with pH1N1 2009, H1N1 2019 or H3N2 2019 virus. Neural co-cultures were mock-treated or inoculated with pH1N1 2009, H1N1 2019 or H3N2 2019 virus with an MOI of 1 at DIV 21. Spontaneous activity was measured every 24 h post inoculation for ten days, and recordings were compared to baseline recording obtained before inoculation at DIV 21. The explanation about all parameters measured for spontaneous activity measured on a single electrode can be found in (**A**). Created in BioRender. 2, V. (2026) https://BioRender.com/m74f1fe. The following variables were displayed: **B** firing rate, **C** burst frequency and **D** burst duration. Data is depicted as mean ± SEM and are derived from at least four independent experiments, with six technical replicates per experiment (n_mock_ = 48; n_pH1N1 2009_ = 48; n_H1N1 2019_ = 24; n_H3N2 2019_ = 24, unless datapoints were excluded based on exclusion criteria (see material and methods). Statistics were tested with a two-way ANOVA test, and corrected for multiple hypothesis testing using the Benjamini–Hochberg method (*q < Q with Q < 0.05, individual *p*-values in Table S3). Asterisks indicate a positive discovery (*)

**Fig. 4:**
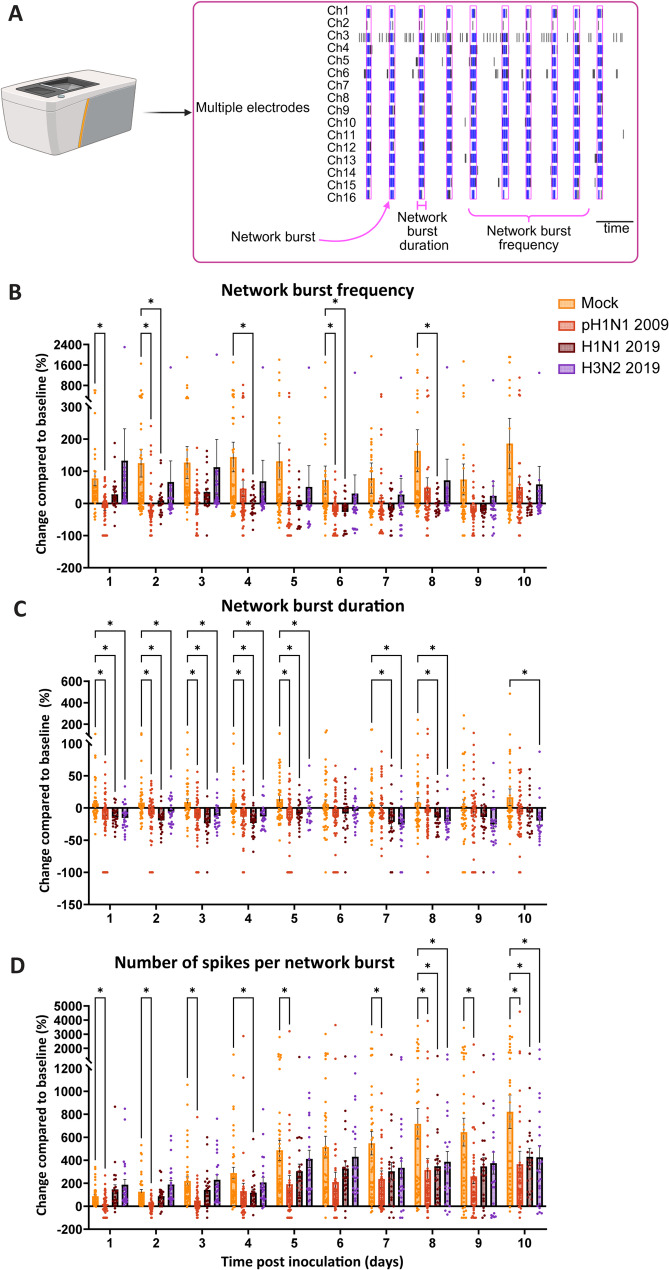
Network activity of neural co-cultures inoculated with pH1N1 2009, H1N1 2019 or H3N2 2019 virus. Neural co-cultures were mock-treated or inoculated with pH1N1 2009, H1N1 2019 or H3N2 2019 virus with an MOI of 1 at DIV 21. Network activity was measured every 24 h post inoculation for ten days, and recordings were compared to baseline recording obtained before inoculation at DIV 21. The explanation about all parameters measured for network activity measured on multiple electrode can be found in (**A**). Created in BioRender. 2, V. (2026) https://BioRender.com/m74f1fe. The following variables were displayed: **B** network burst frequency, **C** network burst duration and **D** spikes per network burst. Data is depicted as mean ± SEM and are derived from at least four independent experiments, with six technical replicates per experiment (n_mock_ = 48; n_pH1N1 2009_ = 48; n_H1N1 2019_ = 24; n_H3N2 2019_ = 24, unless datapoints were excluded based on exclusion criteria see material and methods). Statistics were tested with an two-way ANOVA test, and corrected for multiple hypothesis testing using the Benjamini–Hochberg method (*q < Q with Q < 0.05, individual *p*-values in Table S4). Asterisks indicate a positive discovery (*)

To further investigate the neurovirulent potential of pH1N1 2009, H1N1 2019 and H3N2 2019 viruses on cell viability and neural activity, neural co-cultures were inoculated with pH1N1 2009, H1N1 2019 or H3N2 2019 virus (MOI 1) and neuronal activity was characterized using a MEA system (Axion Systems). The MEA system allows for measuring cell viability and spontaneous activity (Fig. 3A). Cell viability and activity can be analyzed by the number of covered and active electrodes. The number of covered electrodes did not decrease in the inoculated groups compared to the mock controls, indicating no cell death. There was a significant increase in the number of covered electrodes between mock and H1N1 2019 at 2, 5, and 7 to 10 dpi, and between mock and H3N2 2019 at 2 to 6 dpi and 7 to 10 dpi (Figure S4A, two-way ANOVA F (3, 140) = 3.29, *p* = 0.0225, individual *p*-values in Table S3). The number of active electrodes significantly decreased in pH1N1 2009 virus inoculated cultures at 5 to 8, and 10 dpi, H1N1 2019 virus inoculateed cultures at 4 to 8 dpi, and H3N2 2019 virus inocualated cultures at 7 and 10 dpi compared to the mock (Figure S4B, two-way ANOVA F (3, 138) = 3.592, *p* = 0.0154, individual p-values in Table S3). This together suggests that even though the neural cultures are not dying, their activity is affected. Next, we studied the functionality of the neural co-cultures by measuring the spontaneous activity every 24 h after inoculation. Specifically, we analyzed the spontaneous activity that is measured on single electrodes: the firing rate (spikes per time interval), burst frequency (burst per time interval) and burst duration (Fig. 3). In addition, we analyzed the network activity that is measured via multiple electrodes simultaneously: the network burst frequency (network bursts per time duration), network burst duration, and number of spikes per network burst (Fig. 4A). At baseline level before inoculation, there were no differences in the firing rate, burst frequency or network burst frequency between the cultures dedicated for mock or virus inoculation (Fig. S4C, One-way ANOVA F (3, 138) = 0.8416, *p* = 0.4733; Fig. 3D, One-way ANOVA F (3, 134) = 2.082, *p* = 0.1056; Fig. 3E, One-way ANOVA F (3, 131) = 0.1991, *p* = 0.8969, respectively; individual p-values in Table S3). After inoculation, the firing rate was significantly decreased in pH1N1 2009 virus inoculated cultures at 1 to 7 dpi, and 9 dpi, H1N1 2019 virus inoculated cultures at 2, 4 to 10 dpi, and H3N2 2019 virus inoculated cultures at 4 to 10 dpi compared to mock (Fig. 3B; two-way ANOVA F(3, 137) = 5.378, *p* = 0.0016, individual p-values in Table S4). At 1 dpi, there was a significant increase in the firing rate in H3N2 2019 virus inoculated cultures compared to mock (Fig. 3B). As a control, we inoculated neural co-cultures with heat-inactivated pH1N1 2009 to check whether the observed effect was due to the intracellular replication we have observed earlier (Fig. 1B) or due to viral components being present. There was no difference between mock and heat-inactivated pH1N1 2009 virus in the firing rate at 1 or 2 dpi, and the firing rate in pH1N1 2009 inoculated cultures was significantly lower compared to the heat control and mock (Figure S4F, two-way ANOVA F (2, 95) = 28.43, *p* < 0.0001, individual p-values in Table S3). The burst frequency was significantly decreased in pH1N1 2009 virus inoculated cultures at 1 to 3, 5, 6, 8 and 10 dpi, in H1N1 2019 virus inoculated cultures at 4 dpi and in H3N2 virus inoculated cultures 2019 at 2, 4 to 6, 8 and 10 dpi compared to mock (Fig. 3C, two-way ANOVA F (3, 134) = 2.120, *p* = 0.1007, individual p-values in Table S4). The burst duration was also significantly decreased in pH1N1 2009 virus inoculated cultures at 1 to 10 dpi, in H1N1 2019 virus inoculated cultures at 3 to 10 dpi and in H3N2 2019 virus inoculated cultures at 4, 5, 7 to 10 dpi compared to mock (Fig. 3D, two-way ANOVA F (3, 134) = 10.13, *p* < 0.0001, individual *p*-values in Table S4). Additionally, we analyzed the inter-spike-interval (ISI) coefficient of variation (measure of spike regularity), inter-burst-interval (IBI) coefficient of variation (measure of burst regularity), number of spikes per burst and the ISI within burst (measure for burst intensity) (Figure S4G-J, respectively). The ISI coefficient of variation was significantly decreased in pH1N1 2009 virus inoculated culturs at 1 to 10 dpi, in H1N1 2019 virus inoculated cultures at 4 to 6, 8 and 10 dpi and in H3N2 2019 virus inoculated cultures at 5, 8 and 10 dpi compared to mock (Figure S4G, two-way ANOVA F(3, 137) = 7.028, *p* = 0.0002, individual *p*-values in Table S3). There was a significant increase in H3N2 2019 virus inoculated cultures at 1 dpi at the ISI coefficient of variation compared to mock (Figure S4G). The IBI coefficient of variation was significantly decreased in H1N1 2019 virus inoculated cultures at 1 to 5, 7 and 10 dpi, and in H3N2 2019 virus inoculated cultures at 1 to 4 and 10 dpi compared to mock (Figure S4H, two-way ANOVA F (3, 131) = 3.459, *p* = 0.0184, individual *p*-values in Table S3). The number of spikes per burst was significantly decreased in pH1N1 2009 virus inoculated cultures at 1 to 3, 5, 6, 8 and 9 dpi, and in H1N1 2019 virus inoculated cultures at 8 dpi compared to mock (Figure S4I, two-way ANOVA F (3, 134) = 3.455, *p* = 0.0184, individual p-values in Table S3). There was a significant increase in H1N1 2019 virus inoculated cultures at 1 dpi, and in H3N2 2019 virus inoculated cultures at 1 and 2 dpi compared to mock (Figure S4I). The mean ISI within bursts was significantly decreased in H3N2 2019 inoculated cultures at 1 and 3 dpi compared to mock, and a significant increase was observed in pH1N1 2009 virus inoculated cultures at 2 dpi compared to mock (Figure S4J, two-way ANOVA F (3, 134) = 1.032, *p* = 0.3805, individual *p*-values in Table S3).

Analyses of the multiple electrodes’ parameters revealed that the network burst frequency was significantly decreased at 1, 2 and 6 dpi in pH1N1 2009 inoculated cultures compared to mock, and at 2, 4, 6 and 8 dpi in H1N1 2019 inoculated cultures compared to mock (Fig. 4B, two-way ANOVA F (3, 130) = 2.350 *p* = 0.0754, individual *p*-values in Table S5). The network burst duration was significantly decreased in pH1N1 2009 virus inoculated cultures at 1 to 5 dpi, in H1N1 2019 virus inoculated cultures at 1 to 5, 7 and 8 dpi, and in H3N2 2019 virus inoculated cultures at 1 to 5, 7, 8 and 10 dpi compared to mock (Fig. 4C, two-way ANOVA F (3, 130) = 3.479, *p* = 0.0179, individual p-values in Table S5). The number of spikes per network burst was also significantly decreased in pH1N1 2009 virus inoculated cultures at 1 to 3, 5 and 7 to 10 dpi, in H1N1 2019 virus inoculated cultures at 8 and 10 dpi, and in H3N2 2019 virus inoculated cultures at 4, 7 and 10 dpi compared to mock (Fig. 4D, two-way ANOVA F (3, 130) = 5.497, *p* = 0.0014, individual p-values in Table S5).

### Number of synapses increases in pH1N1 2009 inoculated neural co-cultures early during infection

**Fig. 5:**
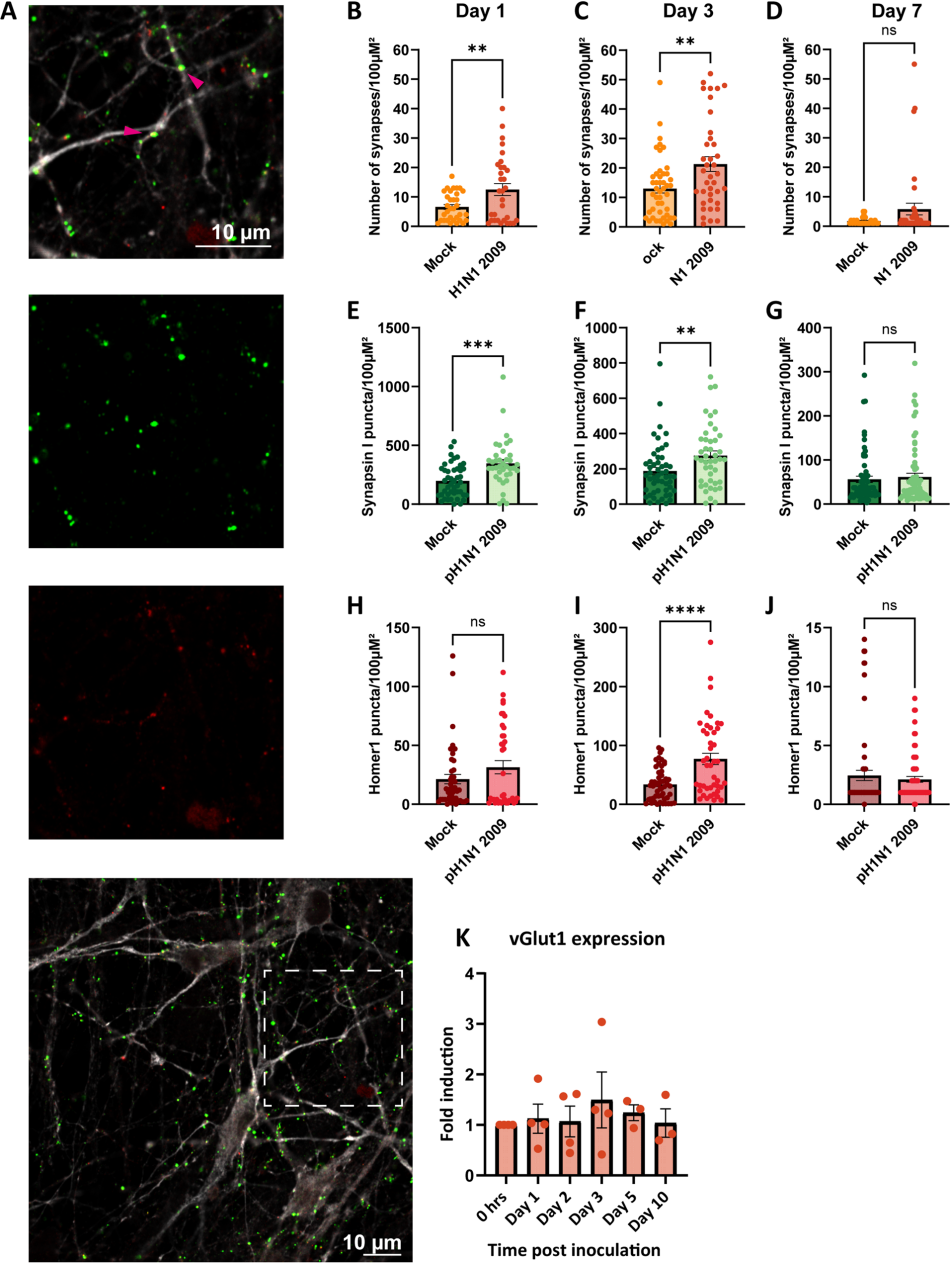
Number of synapses in mock or pH1N1 2009 inoculated cultures. Neural co-cultures were inoculated with pH1N1 2009 (MOI 1), fixed after 1-, 3-, or 7 days post inoculation (dpi), and stained for Synapsin I as a marker for the presynaptic density (green), Homer1 as a marker for the postsynaptic density (red) and with microtubule-associated protein (MAP2; grey) as a marker for neurons. A synapse was counted when co-localization occurred between Synapsin I and Homer1. Example shown in (**A**) with co-localization (yellow) depicted with pink arrows. All data is depicted as mean ± standard error of the mean (SEM), and are derived from at least two independent experiments, with two technical replicates, where at least 15 images were taken from. The number of synapses per high power field is depicted for 1 (**B**), 3 (**C**) and 7 (**D**) dpi. The number of Synapsin I puncta per high power field is depicted for 1 (**E**), 3 (**F**) and 7 (**G**) dpi. The number of Homer1 puncta per high power field is depicted for 1 (**H**), 3 (**I**) and 7 (**J**) dpi. Statistics were tested with an unpaired t-test (equal standard deviation) or Welsh’s t-test (no equal standard deviation). The assumption for equal standard deviations was tested with a Levene’s test. Asterisks indicate statistical significance (ns = not significant, *P < 0.05, **P < 0.01, ***P < 0.001, ****P < 0.0001). (K) Expression of vGlut1 in pH1N1 2009 inoculated neural co-cultures is shown over time. Neural co-cultures were inoculated with a MOI of 1 and lysed after 0, 24, 48 or 72 h and 5- or 10 dpi. Quantitative polymerase chain reaction (qPCR) was performed for β-actin and vGlut1, data is displayed corrected for actin. Data represent mean ± SEM, and are derived from at least three independent experiments, with one biological replicate and two in technical replicates. Statistics were tested with an one-way ANOVA, and corrected for multiple hypothesis testing using the Benjamini–Hochberg method (*q < Q with Q < 0.05, all *p*-values were > 0.05)

We decided to continue additional analysis with pH1N1 2009 virus as the change in spontaneous activity was most prominent (Figs. 3, 4 and S4). Synaptic connections are a necessity for network activity and are refined by neural activity, where connections can be eliminated, maintained or generated. To investigate if a difference in the number of synaptic connections was associated with the observed decrease in neural activity upon inoculation with pH1N1 2009 (Fig. 3), the number of synapses was determined at 1, 3, and 7 dpi. Neural co-cultures were mock-inoculated or with pH1N1 2009 virus, and fixed at 1-, 3- and 7 dpi for immunofluorescence staining for Synapsin I as pre-synaptic marker and Homer1 as post-synaptic marker. We quantified the number of synapses based on co-localization of Synapsin I and Homer1 (example in Fig. 5A; pink arrows). A significant increase in synapses was observed at 1- and 3 dpi in pH1N1 2009 virus inoculated neural co-cultures compared to mock (Fig. 5B, Welch’s t-test t(39.72) = 2.714, *p* = 0.0098; Fig. 5C, Welch’s t-test t(62.04) = 2.834, *p* = 0.0062, respectively). The number of synapses did not significantly differ at 7 dpi (Fig. 5D; unpaired t-test t(62) = 1.703, *p* = 0.0936). We quantified the Synapsin I and Homer1 puncta, to investigate if either or both of those proteins are responsible for the increased number of synapses that we observed. The number of Synapsin I puncta significantly increased at 1 and 3 dpi in pH1N1 2009 virus inoculated cultures compared to mock (Fig. 5E, unpaired t-test t(81) = 4.026, *p* = 0.0001; Fig. 5F, unpaired t-test t(81) = 4.026, *p* = 0.0001, respectively), which was not significant anymore at 7 dpi (Fig. 5G, unpaired t-test t(129) = 0.4984, *p* = 0.6191). The number of Homer1 puncta was only increased at day 3 in pH1N1 2009 inoculated cultures compared to mock (Fig. 5H-G, Welch’s t-test t(56.48) = 4.200, *p* = < 0.0001). In addition, we investigated mRNA levels of the presynaptic glutamate-transporter 1 (vGlut1), an important player in neurotransmission. The expression of vGlut1 can be affected by neuroinflammation [25, 52], for example induced by influenza viruses We did not observe any differences in pH1N1 2009 virus inoculated cultures over time (Fig. 5K, one-way anova F(5, 16) = 0.3286, *p* = 0.8884, individual *p*-values > 0.05).

### Synaptic plasticity is partially impaired after pH1N1 2009 inoculation

Next, we studied synaptic plasticity, which entails the ability to make experience-dependent changes in the strength of neuronal connections, and can be studied by LTP induction. First we induced chemical LTP (cLTP) in mock neural co-cultures at 23 and 31 days of in vitro cultures (DIV) successfully (Figure S5), indicated by an increased local and network synchronization. At DIV 23, the firing rate was significantly decreased between 0.5 and 24 h post induction of cLTP (Figure S5A, two-way ANOVA F (1, 43) = 6.813, *p* = 0.0124; individual p-values in Table S6), and only at 0.5 h post induction of cLTP at DIV 31 (Figure S5B, two-way ANOVA F (1, 22) = 0.5797, *p* = 0,4545; individual p-values in Table S6). At both DIV 23 and 31, the burst frequency was significantly increased at all timepoints post induction of cLTP (Figure S5C, two-way ANOVA F (1, 43) = 86.56, *p* < 0.0001; Figure S5D, two-way ANOVA F (1, 22) = 66.37, *p* < 0.0001, respectively; individual p-values in Table S6), while the burst duration was significantly reduced at the same timepoints (Figure S5E, two-way ANOVA F (1, 43) = 436.4, *p* < 0.0001; Figure S5F, two-way ANOVA, F (1, 22) = 368.3, *p* < 0.0001, respectively; individual *p*-values in Table S6). In addition, the network burst frequency was significantly increased at all timepoints post induction of cLTP (Figure S5G, two-way ANOVA F (1, 43) = 64.79, *p* < 0.0001; Figure S5H, two-way ANOVA F (1, 22) = 101.4, *p* < 0.0001, respectively; individual *p*-values in Table S6), while the network burst duration was significantly reduced at the same timepoints (Figure S5I, two-way ANOVA F (1, 43) = 674.0, *p* < 0.0001; Figure S5J, two-way ANOVA F (1, 22) = 261.8, *p* < 0.0001, respectively; individual p-values in Table S6).

**Fig. 6:**
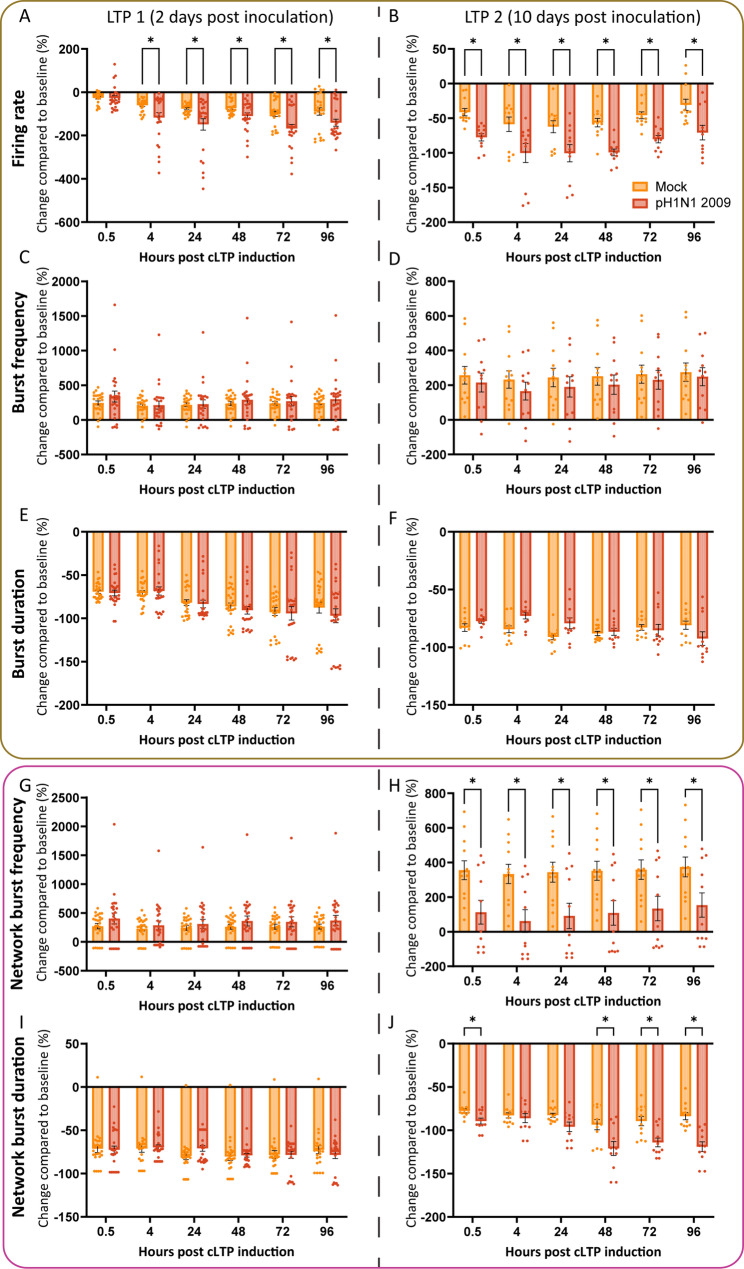
Synaptic plasticity is partially impaired after inoculation with pH1N1 2009. Neural co-cultures were mock-treated or infected with pH1N1 2009 virus with an MOI of 1 at DIV 21. After 2- or 10-days post infection (long-term potentiation 1 (LTP1) and LTP2 respectively), a chemical LTP (cLTP) protocol was employed. Neural activity was measured 0.5-, 4-, 24-, 48-, 72- and 96 h post induction of cLTP. In order to correctly compare the groups, we normalized the data per independent experiment by substracting the group that did not receive cLTP drugs from the group that did receive the cLTP drugs. This way we control for any effects induced by DMSO, and any stress that is put on the neural co-cultures by pipetting and changing in temperature and CO_2_. The following variables were displayed: (**AB**) Firing rate, (**CD**) Burst frequency, (**EF**) Burst duration, (**GH**) Network burst frequency, (**IJ**) Network burst duration. Data is depicted as mean ± SEM and are derived from four independent experiments, with three technical replicates per experiment (n_Mock-2 dpi_ = 24; n_pH1N1 2009–2 dpi_ = 24; n_Mock-10 dpi_ = 12; n_pH1N1 2009–10 dpi_ = 12, unless datapoints were excluded based on exclusion criteria see material and methods). Statistics were tested with an two-way ANOVA test, and corrected for multiple hypothesis testing using the Benjamini–Hochberg method (*q < Q with Q < 0.05, individual *p*-values in Table S6). Asterisks indicate a positive discovery (*)

Next, we wanted to investigate how inoculation with pH1N1 2009 virus (MOI 1) changed the cLTP induction compared to mock neural co-cultures to further investigate the neurovirulent potential of influenza A viruses on synaptic connections. We induced cLTP at 2 and 10 dpi to observe any acute or post-acute effects. In order to correctly compare the groups, we normalized the data per independent experiment by substracting the group that did not receive cLTP drugs from the group that did receive the cLTP drugs. This way we control for any effects induced by DMSO, or stress caused by handeling of the cultures. In the pH1N1 2009 virus inoculated cultures the firing rate was significantly decreased from 4 to 962 h post cLTP induction 2 dpi compared to mock (Fig. 6A, two-way ANOVA F (1, 46) = 9.919, *p* = 0.0029; individual p-values in Table S7). A significant decrease was also observed from 0.5 to 96 h post cLTP induction 10 dpi (Fig. 6B, two-way ANOVA F (1, 21) = 28.31, *p* < 0.0001; individual *p*-values in Table S7). The burst frequency and burst duration did not differ between mock and inoculated cultures at all timepoints post induction of cLTP 2 or 10 dpi (Fig. 6C, two-way ANOVA F (1, 46) = 0.3413, *p* = 0.5619; Fig. 6D, two-way ANOVA F (1, 21) = 0.3620, *p* = 0.5538; Fig. 6E, two-way ANOVA F (1, 46) = 0.2312, *p* = 0.6329; Fig. 6F, two-way ANOVA F (1, 21) = 0.6829, *p* = 0.4179, respectively; individual p-values in Table S7). The network burst frequency did not differ between mock and inoculated cultures post induction of cLTP at 2 dpi (Fig. 6G, two-way ANOVA F (1, 46) = 0.9288, *p* = 0.3402; individual p-values in Table S7). At 10 dpi, the network burst frequency significantly decreased in pH1N1 2009 virus inoculated cultures compared to the mock from 0.5 to 96 h post cLTP induction (Fig. 6H, two-way ANOVA F (1, 21) = 7.450, *p* = 0.0126; individual p-values in Table S7). The network burst duration did not alter between mock and inoculated neural co-cultures post induction of cLTP at 2 dpi (Fig. 6I, two-way ANOVA F (1, 46) = 0.01620, *p* = 0.8993; individual p-values in Table S7), but was significantly decreased at 0.5,- and 48 to 96-h post induction of cLTP at 10 dpi (Fig. 6J, two-way ANOVA F (1, 21) = 10.31, *p* = 0.0042; individual p-values in Table S7). In conclusion, synaptic plasticity is partially impaired 10 days after inoculation with pH1N1 2009 virus, but not in early stages, indicated by a lower response to cLTP measured by network activity**.**

## Discussion

In this study, we revealed that seasonal and pandemic influenza A viruses trigger functional changes in a hiPSC-derived neural co-culture model. Although replication was inefficient, abundant infection of neurons was observed without the induction of cell death or apoptosis. Inoculation of the neural co-cultures with pH1N1 2009 virus, H1N1 2019 or H3N2 virus resulted in a reduction of spontaneous neural activity. Additionally, inoculation of the neural co-cultures with pH1N1 2009 resulted in partially reduced and neural excitability.

Overall, seasonal and pandemic influenza A viruses included in this study did not replicate efficiently or trigger cell death in our neural co-culture model, despite their ability to infect neurons. This aligns with in vivo observations, where seasonal or pandemic influenza A viruses are occasionally detected in the CNS [3, 5, 7–11, 14–21]. However, these viruses do not seem to replicate efficiently in the CNS of mammals [5, 14–22], like observed for highly pathogenic influenza H5N1 viruses [4]. Histological lesions that are associated with active virus replication are not detected in mammals inoculated with seasonal and pandemic influenza A viruses [5, 22]. In vitro studies have shown that seasonal and pandemic influenza A viruses can infect neurons or neuron-like cells [24, 27, 29], which is in accordance with our study. However, in a neuroblastoma cell line, pH1N1 2009 virus was able to replicate efficiently [27], which we did not observe in our hiPSC-derived neural cultures. In general, there are large differences in the replication efficiency between influenza A viruses in neural cells. Highly pathogenic influenza H5N1 viruses replicate more efficiently in cells of the CNS both in vitro and *in vivo* [53–55] compared to seasonal and pandemic influenza A viruses. Sialic acid binding preferences, the multibasic cleavage site or changes in the polymerase genes are suggested to be associated with these differences in neurotropism [4, 27]. Since the replication of influenza A viruses is multifactorial, the exact role of viral and host factors that play a role in the neurotropism of influenza A viruses remains to be elucidated.

Influenza A virus infection reduces spontaneous neural activity and reduces partially excitability in a time-dependent manner. This aligns with a decrease in spontaneous neurotransmission that was observed in mouse-adapted influenza A virus infected mouse hippocampal neurons by whole-cell patch clamping [51]. In our study, neuronal excitability—measured after cLTP stimulation—was only impaired at the level of the network burst frequency. A reduction in neuronal excitability, using paired-pulse stimulation or evoked via current clamp, was also observed in mouse-adapted influenza A virus infected mouse hippocampal neurons, which recovered over time [23, 51]. In general, there are many mechanisms which can play a role in a reduction of spontaneous neuronal activity or excitability, including cell death, senescence, cytokines and changes in spines or synapses, neurotransmitter release or ion channels. Virus-induced changes can for example be dendritic loss and an impaired glutamate stimulation response, which was observed after Herpes Simplex Virus 1 infection [56], or altered synaptic activity after SARS-CoV-2 infection [57]. In contrast, neurovirulent cytokines, elicited by a mouse neuroinvasive coronavirus, increased neuronal excitability in mouse neurons [58]. The increased number of synapses, where a loss of synapses was expected based on the reduction in neurotransmission fits with the observed reduction in the complexity of dendritic spines in a mouse model [23]. We speculate a compensatory response to the reduction of neurotransmission resulted in the increase of synaptic density markers in our model, which would fit with the comparable neurotransmission pattern between the inoculated and mock cultures later in infection. The mechanism by which influenza A virus infection leads to reduced neuronal activity and excitability remains unknown, as it does not appear to involve cell death, synapse loss, or reduced vGlut1 mRNA expression in our study.

Impaired synaptic plasticity is strongly correlated with cognitive impairments, like learning and memory problems. Influenza A virus infection causes cognitive impairments in human cases and experimentally inoculated mice [2, 23, 41, 59]. Impairments in spatial learning and memory, search strategy and cognitive flexibility were observed in H3N2- and H7N2 virus inoculated mice in a time-dependent manner [23]. H1N1 WSN virus-inoculated mice showed long-term behavioral changes of anxiety and cognition [59]. The observed reduction of spontaneous neural activity and excitability in pH1N1 2009 virus inoculated neural co-cultures could play a role in behavior and cognitive impairments as shown by Hosseini et al. [23]. The effects of viral infections, like influenza A viruses, on behaviour and cognitive problems is also highlighted in a recent study where non-severe respiratory infections by SARS-CoV-2 and influenza A virus are associated with a similar risk of post-acute sequelae, including neurological symptoms and neurodegenerative disease [44, 60].

We present here a scalable, human-derived model in which we can study the neurotropism and neurovirulent potential of viral infections on a functional level. We show in a human system that –despite the lack of abundant replication and cell death- influenza A virus infection results in neural dysfunction that could contribute, at least in part, to the development of cognitive problems observed in humans. This is important because there is limited knowledge on the neurotropic and neurovirulent potential of a wide range of viruses that are not classically considered as neurotropic or neurovirulent, such as influenza A viruses and SARS-CoV-2. Consequently, the brain is often overlooked while researching these viruses. In addition, so far most in vitro studies have used either neuronal cell lines and/or lab-adapted or mouse-adapted viruses, which tend to be more neuropathogenic and therefore do not resemble the neuropathology in human cases.

Altogether, our findings reveal that pandemic and seasonal influenza A viruses do not replicate efficiently in a hiPSC-derived neural co-culture model. Even though replication is limited, recent seasonal and pandemic influenza A viruses are neurovirulent in this model, characterized by changes in neuronal functioning, like spontaneous neurotransmission and excitability. The insidious changes that we observe in our model can likely contribute to acute and post-acute sequelae of influenza.

## Materials and methods

### Cells

MDCK (ATCC) cells were cultured in Eagle minimal essential medium (EMEM; Capricorn scientific) supplemented with 10% fetal calf serum (FCS; Sigma), 100 IU/mL penicillin (ThermoFisher Scientific), 100 µg/mL streptomycin (ThermoFisher Scientific), 2 mM glutamine (ThermoFisher Scientific), 1.5 mg/mL sodium bicarbonate (ThermoFisher Scientific), 10 mM HEPES (ThermoFisher Scientific) and 0.1 mM non-essential amino acids (ThermoFisher Scientific). MDCKs were passaged when confluency was over 90%. First, cells were washed with phosphate-buffered saline (PBS) and then dissociated with trypsin–EDTA (0.05%). Medium was refreshed once a week, and cells were kept at 37 °C and 5% CO_2_. Cells were routinely tested for mycoplasma contamination.

### Human induced pluripotent stem cells

Human induced pluripotent stem cells (hiPSCs; WTC-11 Coriell no. GM25256, provided by Bruce R. Conklin, The Gladstone Institutes and UCSF, San Francisco, CA, USA) were used to generate neural co-cultures. hiPSCs were plated on Matrigel-coated plates (Sigma, 10 µl/mL, resuspended in knockout Dulbecco’s Modified Eagle Medium, KO DMEM; ThermoFisher Scientific) and maintained in hiPSC medium (Table 1). hiPSCs were passaged when confluency of 70–80% was reached. For passaging, hiPSC were washed with PBS and released with Accutase (Life Technologies). Medium was refreshed every other day, and cells were cultured at 37 °C and 5% CO_2_.

**Table 1:** Overview of mediums used for hiPSC-derived NGN2 neurons and astrocytes

Name	Reagents with final concentration	Manufacturer
hiPSC medium	Stemflex medium	ThermoFisher Scientific
	100 IU/mL penicillin	ThermoFisher Scientific
	100 µg/mL streptomycin	ThermoFisher Scientific
	10 µl/mL RevitaCell	ThermoFisher Scientific
Differentiation medium	Advanced DMEM/F12 medium	ThermoFisher Scientific
	100 IU/mL penicillin	ThermoFisher Scientific
	100 µg/mL streptomycin	ThermoFisher Scientific
	0.1 mM non-essential amino acids	ThermoFisher Scientific
	1% N2 supplement	ThermoFisher Scientific
	10 ng/mL Human Recombinant Neurotrophin-3 (NT3)	Stemcell Technologies
	10 ng/ml brain-derived neurotrophic factor (BDNF)	Prospecbio
	200 ng/mL laminin	Sigma Corning
	4 µg/mL doxycycline	Sigma
NGN2 medium	Neurobasal medium	ThermoFisher Scientific
	100 IU/mL penicillin	Lonza
	100 µg/mL streptomycin	Lonza
	2 mM glutamine	Lonza
	2% B27 minus RA supplement	ThermoFisher Scientific
	10 ng/mL Human Recombinant Neurotrophin-3 (NT3)	Stemcell Technologies
	10 ng/mL brain-derived neurotrophic factor (BDNF)	Prospecbio
	4 µg/mL Doxycycline (DOX)	Sigma
Neural progenitor cells medium	Advanced DMEM/F12 medium	ThermoFisher Scientific
	100 IU/mL penicillin	Lonza
	100 µg/mL streptomycin	Lonza
	1% N2 supplement	ThermoFisher Scientific
	2% B27 minus RA supplement	ThermoFisher Scientific
	1 µg/mL laminin	Sigma-Aldrich Corning
	20 ng/mL Fibroblast Growth Factor (FGF)	Merck
Astrocytes medium	Advanced DMEM/F12 medium	ThermoFisher Scientific
	100 IU/mL penicillin	Lonza
	100 µg/mL streptomycin	Lonza
	1% N2 supplement	ThermoFisher Scientific
	2% B27 minus RA supplement	ThermoFisher Scientific
	1 µg/mL laminin	Sigma-Aldrich Corning
	10 ng/mL Bone Morphogenetic Protein 4 (BMP4)	Prospec
	10 ng/mL Leukemia Inhibitory Factor (LIF)	Tebu-bio

### Differentiation of hiPSCs into NGN2 neuron-astrocyte co-cultures

hiPSCs were differentiated into excitatory cortical neurons by overexpression of neuronal determinant NGN2 via tetracycline-controlled transcriptional activation as previously described [61, 62]. In short, coverslips (1.5H, 0117530; Paul Marienfeld) or plates were coated with poly-L-ornithine (Sigma, 100 µg/mL) for 1 h at RT in the dark. Then coverslips were washed three times with sterile water and air-dried for 1 h. Coverslips or plates were then coated with either laminin (Sigma & Corning, 50 µl/mL, resuspended in DMEM, ThermoFisher Scientific) or Matrigel (Corning, 10 µl/mL resuspended in KO DMEM) for 1 h at 37 °C. After incubation, the hiPSCs were plated in hiPSC medium (Table 1) supplemented with 4 µg/mL fresh doxycycline (Sigma). The next day, the medium was refreshed with differentiation medium (Table 1). In every medium, growth factors were added (fresh) before refreshing. In order to guarantee the formation of functional synapses and thus functional synaptic plasticity within the network, hiPSC-derived astrocytes are needed for the NGN2 neurons to mature [61, 62]. Therefore, on day 3, hiPSC-derived astrocytes were added to the culture in a 1:1 ratio (75.000 neurons: 75.000 astrocytes for 24-well plates surface area; 20.000 neurons: 20.000 astrocytes for 96-well plate surface area). hiPSC-derived astrocytes were differentiated as described previously from hiPSC-derived neural progenitor cells (NPCs) [63]. During maintainance, and differentiation of hiPSC-derived NPCs and astrocytes, medium was refreshed with astrocyte or NPC medium (Table 1) every other day. Cells were were split when confluent, roughly once per week. On day 4, medium of the co-cultures was refreshed with NGN2 medium (Table 1). During the differentiation and maturation of neural co-cultures, half of the medium was refreshed every other day. After DIV 21, the neural co-cultures were used in experiments. All cells were kept at 37 °C and 5% CO_2_.

### Viruses

Pandemic 2009 H1N1 virus (pH1N1 2009, A/Netherlands/602/2009) was isolated from a patient who visited Mexico and the virus was propagated twice in MDCK cells. Seasonal H1N1 virus and H3N2 virus isolated from 2019 were kindly provided by the National influenza centrum (Table 2) and the viruses were propagated once in MDCK cells. Virus stocks were clarified by centrifugation at high speed to remove residual cellular debris prior to aliquoting and storage, and were kept at -80 °C until usage. As a control, heat-inactivated pH1N1 2009 virus was included. Virus was heat-inactivated at 56 °C for 30 min before usage as described by others [64, 65]. All experiment with infectious pH1N1 2009, H1N1 2019 and H3N2 virus were performed in a class II biosafety cabinet under biosafety level 2 (BSL-2) conditions at the Erasmus Medical Center.

**Table 2:** Subtype and clade of 2019 viruses and 2009 virus

Virus	Subtype	Clade	Dutch identification number
H3N2 2019	AH3N2	3C.3a	19A01618
H1N1 2019	AH1N1pdm09	6B.1A5A	19A01659
H1N1 2009	AH1N1pdm09	6B.1	GISAID Isolate ID: EPI_ISL_31217

### Virus infection

Before infection, medium was removed and cells were inoculated with pH1N1 2009, H1N1 2019 or H3N2 2019 viruses a with MOI of 1. Viruses were diluted in NGN2 medium (Table 1) for neural co-cultures, and in infection medium for MDCKs (EMEM supplemented with 100 IU/mL penicillin, 100 µg/mL streptomycin, 2 mM glutamine, 1.5 mg/mL sodium bicarbonate, 10 mM HEPES, 0.1 mM nonessential amino acids, and 1 µg/µL TPCK-treated trypsin (Sigma)). After 1 h of infection MDCK cells were washed three times to remove residual unbound virus. For infection of the hiPSC-derived neural co-cultures, virus-containing medium was replaced with fresh NGN2 medium (Table 1), without washing. The new medium was mixed with old medium that was taken off before inoculation 1:1 to ensure that important nutrients were still present.

### Virus titration

Virus titers were determined by endpoint dilution on MDCK cell as described before [66]. In short, tenfold serial dilutions in triplicates of cell supernatant were prepared in infection medium for MDCKs. MDCKs were washed once with plain EMEM to remove residual FCS prior to adding the diluted supernatants to the MDCKs. After 1 h incubation period at 37 °C and 5% CO_2_, inoculum was removed and fresh infection medium was added to the cells. The supernatants of the infected MDCKs were tested for agglutination three days after inoculation. For that, supernatant was mixed with 0.33% turkey red blood cells and incubated for one hour at 4 °C. The titers of infectious virus were calculated according to the method of Spearman and Kärber [67, 68], and expressed as TCID_50_/mL.

### RNA isolation, qRT-PCT, cDNA production and qPCR

Influenza A virus RNA was detected in samples using a quantitative reverse-transcription polymerase chain reaction (qRT-PCR) assay targeting the M gene segment as described previously [69]. In short, RNA was isolated by using a MagnaPure LC system with a MagnaPure LC total nucleic acid isolation kit (Roche Diagnostics). The concentration of RNA was determined using a NanoDrop spectrophotometer. Influenza A virus was detected by targeting the matrix gene (M; Table 3). The PCR program was as follows: (2 min 50 °C, 20 s 95 °C followed by 45 cycli of 3 s 95 °C and 30 s 60 °C). Reactions were performed on a 7500 Real-Time PCR system (Applied Biosystems).

**Table 3:** Primer-probes targeting the matrix gene of influenza A virus

Influenza A virus (matrix gene)	Infa_fw	CTTCTRACCGAGGTCGAAACGTA
	Infa_rev	TCTTGTCTTTAGCCAYTCCATGAG
	Infa_probe 1	FAM-TCAGGCCCCCTCAAAGCCGAGA-BHQ-1
	Infa_probe 2	FAM-TCAGGCCCCCTCAAAGCCGAAA-BHQ-1

500 ng RNA was reverse transcribed into cDNA using the SuperScript IV reverse transcriptase (Invitrogen) according to the manufacturer’s protocol. Subsequently, the presence of vGlut1 was evaluated by qPCR (Table 4), on a 7500 Real-Time PCR system (Applied Biosystems). Fold changes were calculated using the 2^−ΔΔCt^ method. Normalization was performed using the mean Ct values of household gene Actin as a loading control for every sample.

**Table 4:** Gene-specific primers for vGlut1 and Actin

β-actin_fw	5′-CCCTGGACTTCGAGCAAGAG-3′
β-actin_rev	5′-ACTCCATGCCCAGGAAGGAA-3′
vGlut1_fw	5′-GAGGAGTGGCAGTACGTGTTCC-3′
vGlut1_rev	5′-TCTCCAGAAGCAAAGACCCC-3′

### Immunofluorescence staining

Cells were fixed using 10% formalin for 30 min and washed afterwards with PBS. Cells were permeabilised for 15 min using 1% Triton X-100 in PBS and blocked with in PBS supplemented 0.5% Triton X-100 and 1% bovine serum albumin for 30 min at RT. Primary antibodies (Table 5) incubation was performed for 1 h at RT in blocking solution, except for α-Homer1, α-Synapsin I, which were incubated O/N at 4 °C. After washing with blocking solution, secondary antibodies conjugated to Alexa-488, Alexa-555, Alexa-647 (Invitrogen, Table 5) were used with the corresponding primary antibody and incubated for 1 h at RT in blocking solution. Slides were washed in blocking solution and incubated with a solution of Hoechst (ThermoFisher Scientific) in blocking buffer for 15 min. Slides were again washed in blocking solution, dipped in water and mounted in ProLong Glass Antifade Mountant (ThermoFisher Scientific). Samples were imaged using a Zeiss LSM 700 confocal microscope and synapses were images using a Zeiss LSM 900 confocal microscope.

**Table 5:** List of used antibodies and according dilutions

Antibodies	Manufacturer	Dilutions	Final concentration
Mouse α-NP	EVL, EBS-I-047, Clone Hb65	1:1000	2.0 µg/mL
Rabbit α-GFAP	Millipore, AB5804	1:200	0.5% v/v
Guinea Pig α-MAP2	Synaptic Systems, 188,004	1:200	0.5% v/v
Rabbit α-cleaved caspase 3	Cell Signalling, 9661	1:200	0.5% v/v
Mouse α-Homer1	Synaptic systems, 160,011	1:75	13.33 µg/mL
Rabbit α-Synapsin I	Synaptic systems, 106,103	1:100	10 µg/mL
Hoechst	ThermoFisher Scientific, 62,249	1:5000	0.2 µg/mL
Donkey α-mouse A488	Invitrogen, A21202	1:200	10 µg/mL
Donkey α-rabbit A555	Invitrogen, A31572	1:200	10 µg/mL
Goat α-guinea pig A647	Invitrogen, A21450	1:200	10 µg/mL

### Pixel-based quantification for infection and cleaved caspase-3 staining

To quantify infection percentage, 5 images were taken from coverslips from three independent experiments. The staining of GFAP or MAP2 was annotated per image using the pixel classifier function in QuPath 0.6.0 [70] with a Gaussian filter. The threshold values to determine positive surface area can be found in Table 6. The positive area was then annotated and from this annotation the pixel classifier function was run again to determine the positive area of virus staining (NP). Individual data points were plotted with mean ± SEM.

**Table 6:** Chosen threshold values used for pixel-based quantification in QuPath to determine positive areas

Experiment	Timepoint	Staining (fluorophore)	Threshold
Infection	Day 3, 5 and 10	NP (Alexa488)	40
	Day 3 and day 5	GFAP (Alexa555)	30
	Day 3 and day 5	MAP2 (Alexa647)	25
	Day 10	GFAP (Alexa555)	40
	Day 10	MAP2 (Alexa647)	35
Cleaved caspase-3	Day 3	NP (Alexa488)	33
	Day 3	CC3 (Alexa555)	35
	Day 3 and day 5	MAP2 (Alexa647)	35
	Day 5	NP (Alexa488)	40
	Day 5 and day 10	CC3 (Alexa555)	40
	Day 10	NP (Alexa488)	40
	Day 10	MAP2 (Alexa647)	35

To quantify the expression of cleaved caspase-3, 5 images were taken from coverslips from three independent experiments. The staining area of MAP2 was determined by annotating the positive area that was measured with the pixel classifier function in QuPath 0.6.0 (threshold values in Table 6). The positive area of cleaved caspase-3 was then determined using the pixel classifier function in QuPath 0.6.0 (threshold values in Table 6) on the annotated area of MAP2. Individual data points were plotted with mean ± SEM.

### Microscopic analysis for synapses

Neural co-cultures were mock-inoculated or inoculated with pH1N1 2009 as described earlier and fixed after 24-, 72 hpi or 7 dpi. Neural co-cultures were stained for Homer1 and Synapsin I as described earlier to quantify the number of synapses. Confocal images were taken with a Zeiss LSM 900 module on an Axio Imager Z2 upright microscope fitted with a 63 × 1.4NA plan-apochromat oil objective. For imaging the Hoechst, Alexa Fluor 488, Alexa Fluor 555 and Alexa Fluor 647 dies, samples were excited using a 405 nm, 488 nm, 561 nm and 640 nm diode laser respectively. Emission was filtered appropriately for each die with 400–600 nm, 410–546 nm (SP545), 540–617 nm, 645–700 nm filter ranges, respectively. ZenBlue image acquisition software (Carl Zeiss gmbh Oberkochen) was used to capture all images. Pixelsize was set to 319.5 µm × 319.5 µm for optimal resolution (1024 × 1024 pixels). Per timepoint at least 15 images were taken per coverslip from two independent experiments. Using of FIJI (version 1.54f), puncta of Synapsin I and Homer1 were selected by the function Find Maxima. A synapse was counted when co-localization occurred between Synapsin I and Homer1. We used a FIJI plugin (‘Nearest Neighbours’) available at https://github.com/ErasmusOIC/NearestNeighbourAnalysis to determine the distance between each Homer1 puncta and its nearest Synapsin I neighbour. Subsequently we defined puncta as co-localizing if the distance between the Homer1 and Synapsin I was below 250 nm.

### MEA recordings of spontaneous neural activity in NGN2 neurons co-cultured with astrocytes

For MEA recordings, hiPSC-derived neural co-cultures were plated onto 24-well MEA plates (Axion Biosystems) as described before. Plates were acclimatized for > 10 min before spontaneous activity was recorded for 3 min every 24 h after inoculation using the Axion Biosystems Maestro MEA at 37 °C and 5% CO_2_. Data analysis was performed using AxIs software (Axion Biosystems Inc.). Covered electrodes were defined as electrodes with a minimum resistance of 18 kΩ. We excluded wells for further analysis if ≤ 5 electrodes were covered. Active electrodes were defined as electrodes with a minimum of five spikes per minute. We excluded wells for analysis if a well had ≤ 5 active electrodes at the baseline recording. The threshold for spike detection was defined as ≥ sixfold the standard deviation (SD) of the root mean square noise. The threshold for burst detection was defined as > 5 spikes within a time window of 100 ms. The threshold for network burst detection was defined as > 50 spikes within a time window of 100 ms, with a minimum of 50% participating electrodes per well.

### Induction of chemical long-term potentiation in NGN2 neurons co-cultured with astrocytes

We employed a cLTP protocol demonstrated earlier in human iPSC-derived neuronal network cultures [71] to study synaptic plasticity in our NGN2 neurons co-cultured with astrocytes. In short, forskolin (Tocris; final concentration 50 μM) and rolipram (Tocris; final concentration 0.1 μM) were dissolved in DMSO and further diluted in NGN2 medium. A maximum amount of 50 µL diluted drug mixture were added to the well, not exceeding a total of 500 µL (Table 1). As negative control, the same amount of DMSO was diluted in NGN2 medium (Table 1). These mixtures were pre-warmed for at least 15 min at 37 °C. A baseline recording was performed before inducing cLTP. The drug mixtures were added directly to the medium, and neural activity was measured after 30 min of incubation. After incubation, drugs were washed out as follows: 100 µl of fresh medium per well was removed and 100 µl of fresh medium per well was added, this was repeated three times. Then 150 µl of fresh medium per well was removed and 150 µl of fresh medium per well was added, this was repeated two times. After wash-out, we recorded neural activity after 4, 24, 48, 72 and 96 h. Settings for MEA recordings and analysis were done exactly the same as for the spontaneous neural activity recordings. Data was normalized based on the average values of the negative control wells (-cLTP group) per independent experiment to control for solvent effect, and any perturbations that are caused by changes in temperature or CO_2_, and pipetting.

### Multiplex bead assay for cytokine profiling

Cytokines were measured using the LEGENDplex™ human antivirus response or human neuroinflammation panel (BioLegend). The kit was used according to the manufacturer’s manual. Concentration was determined with a BD FACS Lyric. Data was analysed with the LEGENDplex™ Data Analysis Software Suite.

### Statistical analysis

Statistical differences between experimental groups were determined as described in the figure legends and text. *P* values of ≤ 0.05 were considered significant. Where necessary, correction for multiple-hypothesis testing was performed using the Benjamini–Hochberg method. Graphs and statistical tests were made with GraphPad Prism version 10.6.1. All individual p-values are supplemented as supplementary tables. Figures were prepared with ImageJ 1.54p and Adobe Illustrator 27.8.1.

## Supplementary Information

Below is the link to the electronic supplementary material.

Supplementary Material 1

## Data Availability

No datasets were generated or analysed during the current study.
