# Supplementary material for: Influenza A virus infection impairs neuronal activity in human iPSC-derived NGN2 neural co-cultures
Source: Acta Neuropathol Commun. 2026 Apr 18;14:121. doi: 10.1186/s40478-026-02292-0 (PMC13235118; doi:10.1186/s40478-026-02292-0)
Supplement: Supplementary file 1 — Supplementary Material 1 [file 40478_2026_2292_MOESM1_ESM.docx]

**Supplementary Information**

**Influenza A Virus Infection Impairs Neuronal Activity in Human iPSC-Derived NGN2 Neural Co-Cultures**

*Authors*: Feline F. W. Benavides^1^, Annabel L. V. Kempff^1^, Hilde Smeenk^2^, Bas Lendemeijer^2,4^, Marla Lavrijsen^3^, Johan A. Slotman^3^, Steven A. Kushner^4,5^, Femke M. S. de Vrij^2^, Lisa Bauer^1^, Debby van Riel^1*^*­­­*

*Affiliations*: ^1^Department of Viroscience, Erasmus MC, Rotterdam, the Netherlands,

^2^Department of Psychiatry, Erasmus MC, Rotterdam, The Netherlands

^3^Optical Imaging Centre and department of Pathology, Erasmus MC, Rotterdam, The Netherlands

^4^Stavros Niarchos Foundation (SNF) Center for Precision Psychiatry & Mental Health, Columbia University, New York, NY, USA,

^5^Department of Psychiatry, Columbia University Irving Medical Center, New York, NY, USA

*E-mail: d.vanriel@erasmusmc.nl

**
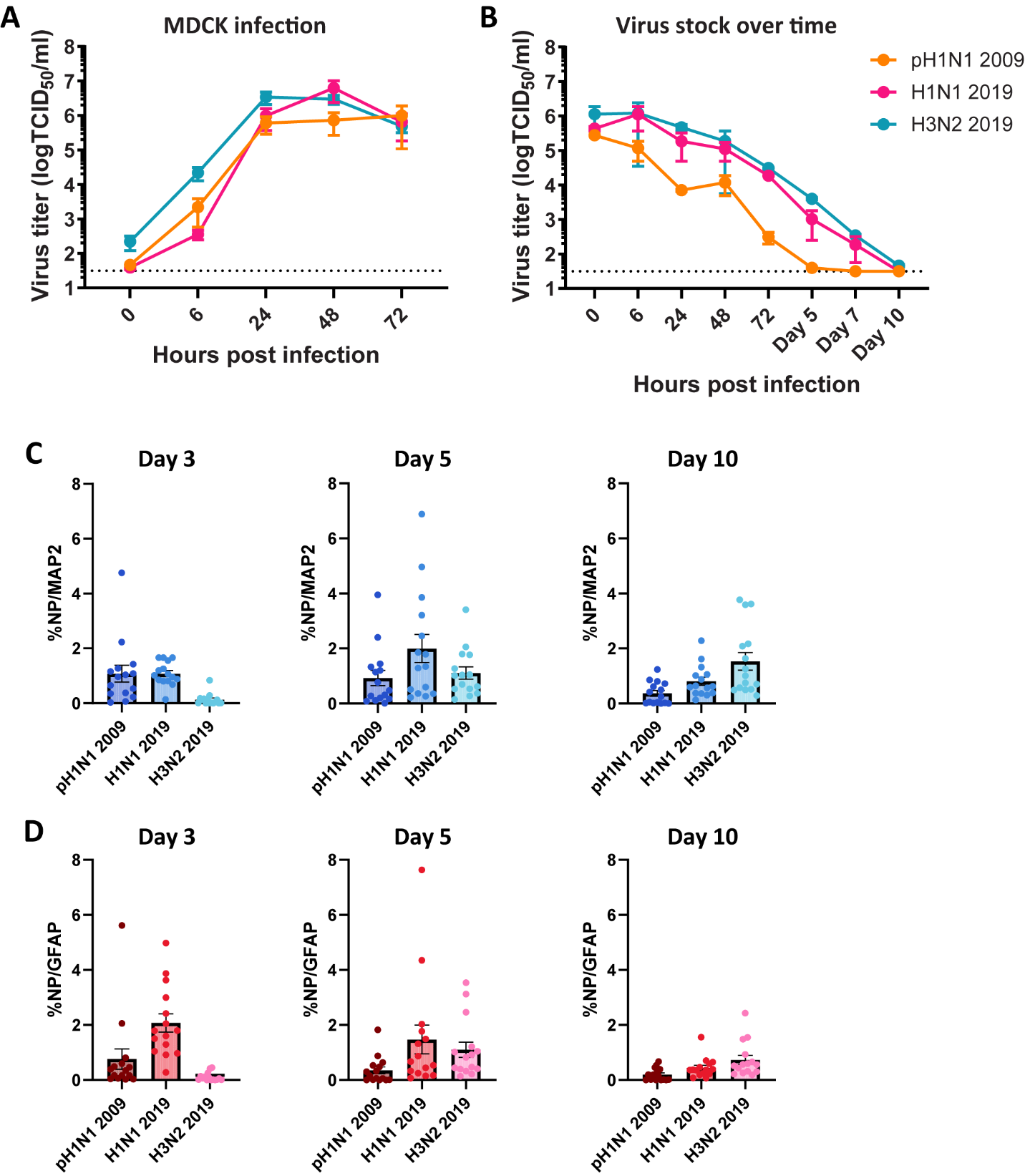
Fig S1. Replication of pH1N1 2009, H3N2 2019 and H1N1 2019 virus in MDCK cells and without cells, and infection quantification in neural co-cultures.** (A) Madin-Darby Canine Kidney (MDCK) cells were inoculated in parallel with neural co-cultures with pH1N1 2009, H1N1 2019 and H3N2 2019 with a MOI of 1. Data represent mean ± standard deviation (SD) and are derived from three independent experiments, with three biological replicates and three technical replicates. Dotted line represents lower limited of detection. (B) Virus stocks of pH1N1 2009, H1N1 2019 and H3N2 2019 virus were monitored over time without cells to study the stability of the viruses over time at 37°C and 5% CO_2_. Data represent mean ± SD and are derived from three independent experiments, with three biological replicates and three technical replicates. Dotted line represents lower limited of detection. (C) Pixel-based quantification was performed to detect the nucleoprotein (NP)^+^ signal over the microtubule-associated protein (MAP2)^+^ signal at 3, 5 and 10 days post inoculation (dpi). (D) Pixel-based quantification was performed to detect the NP^+^ signal over the glial fibrillary acidic protein (GFAP)^+^ signal at 3, 5 and 10 dpi.

**
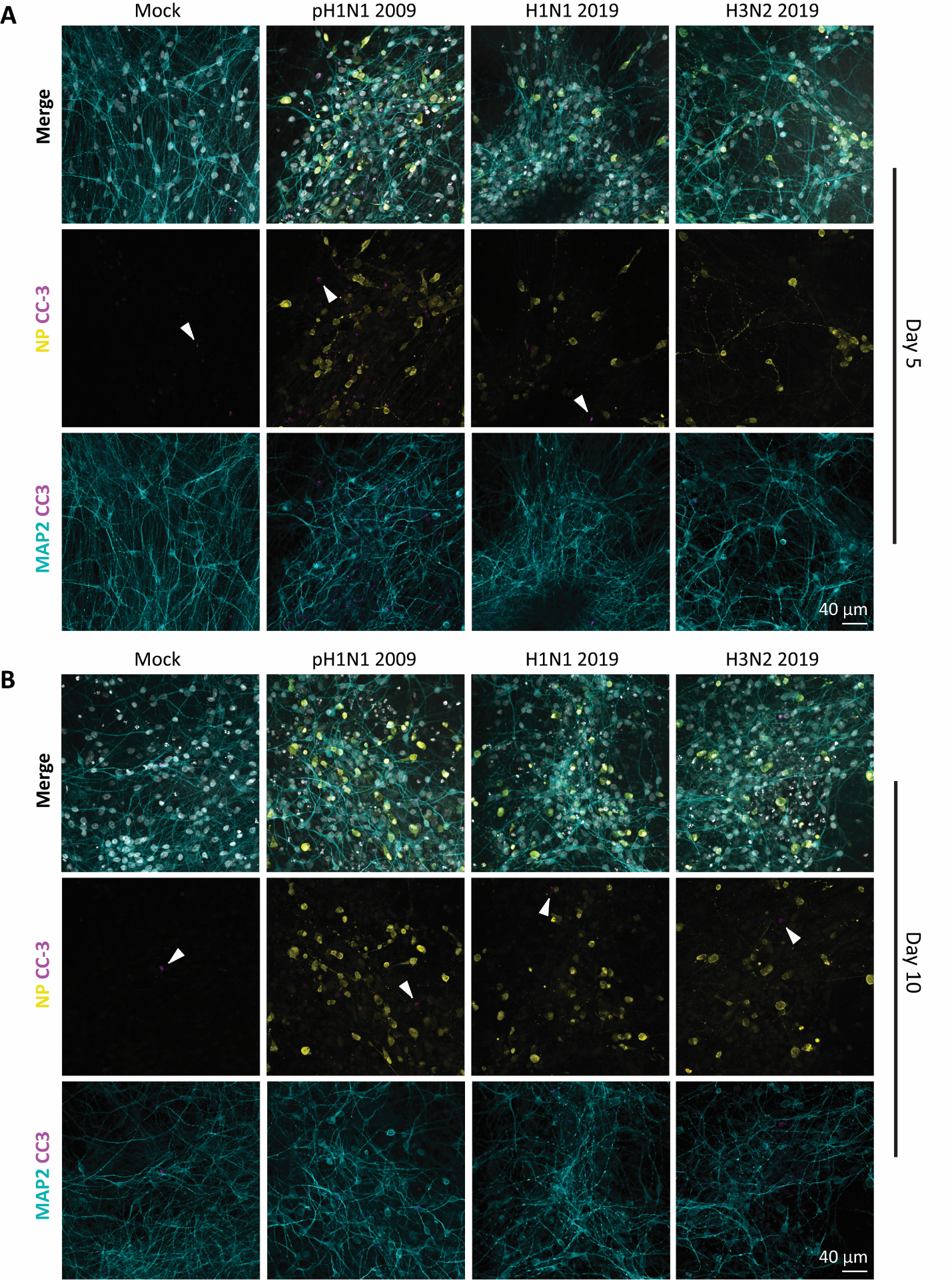
Figure S2. No induction of the apoptotic marker cleaved caspase-3 in the neural co-cultures inoculated with pH1N1 2009, H1N1 2019 or H3N2 2019 virus.** Neural co-cultures were fixed (A) 5 days post infection (dpi) or (B) 10 dpi and were stained with microtubule-associated protein (MAP2; cyan) as a marker for neurons, cleaved caspase-3 (CC-3; magenta, indicated with white arrows) as a marker for apoptosis, and influenza A virus nucleoprotein (NP; yellow) to identify infected cells. Cells were counterstained with Hoechst (grey) to visualize the nuclei. Data shown are representative examples from three independent experiments.

**
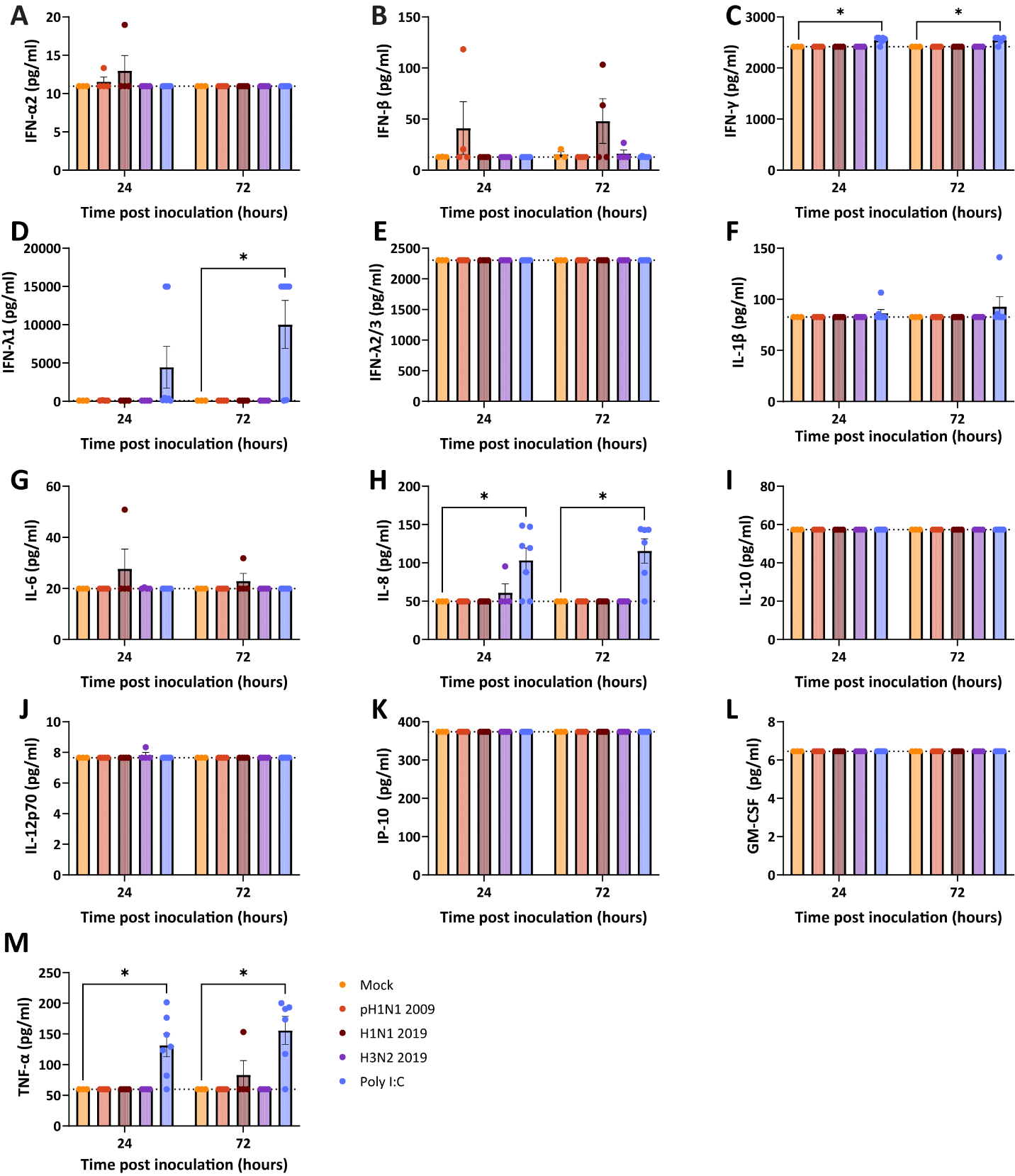
Figure S3. No difference in immune responses in mock neural co-cultures compared to pH1N1 2009, H1N1 2019 or H3N2 2019 virus inoculated cultures.** Neural co-cultures were inoculated with pH1N1 2009, H1N1 2019 or H3N2 2019 (MOI 1), at 24 and 72 hours post inoculation supernatants were taken and cytokine/chemokine release was measured. Data is depicted as mean ± SEM and are derived from three independent experiments, with at least one technical replicate. Statistics was tested with a two-way ANOVA test, and corrected for multiple hypothesis testing using the Benjamini-Hochberg method (*q<Q with Q<0.05, individual p-values in Table S2).

**
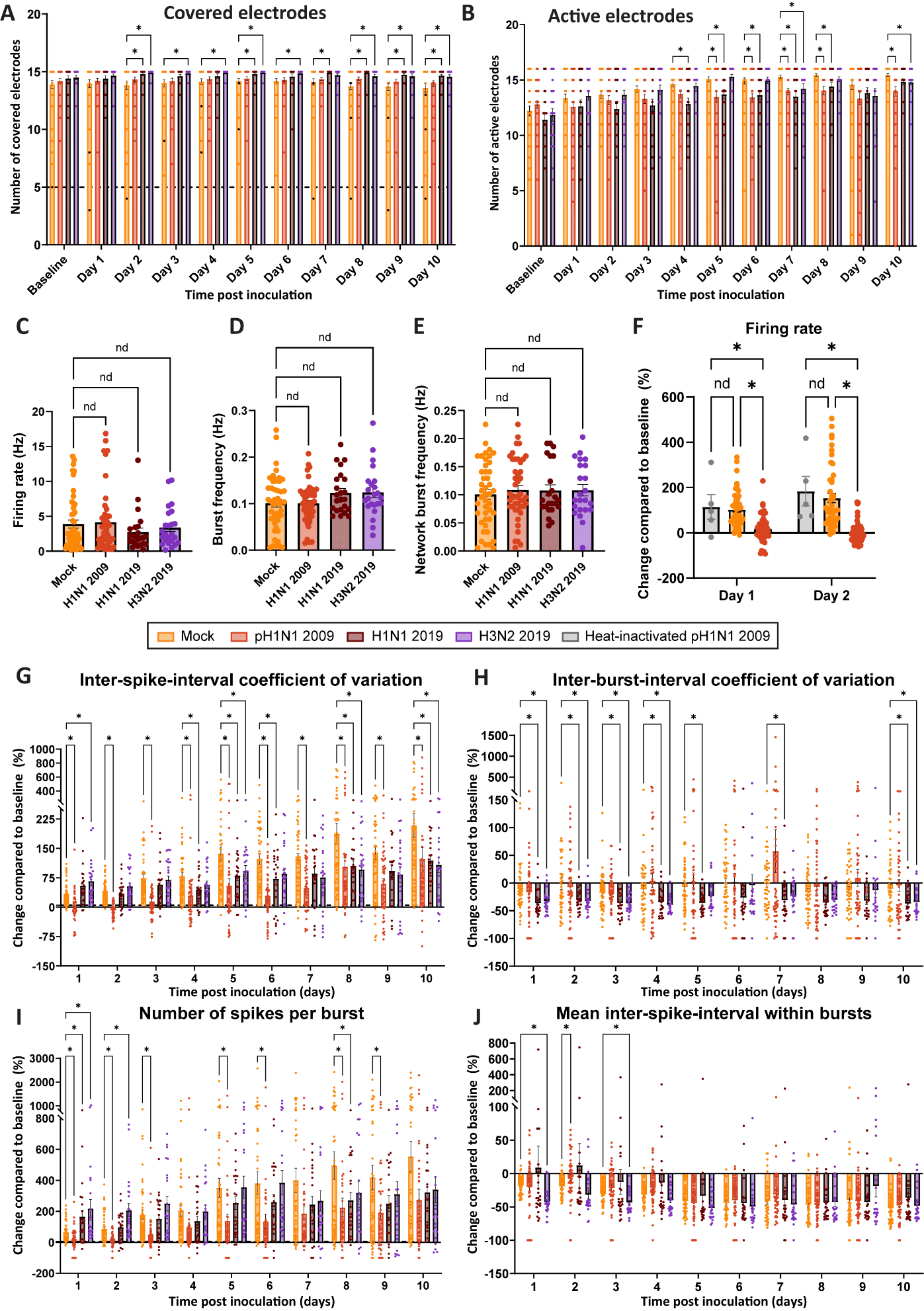
Figure S4. Single electrode spontaneous activity measured of neural co-cultures inoculated with pH1N1 2009, H1N1 2019 or H3N2 2019 virus.** Neural co-cultures were mock-treated or inoculated with pH1N1 2009, H1N1 2019 or H3N2 2019 virus with an MOI of 1 at DIV 21. Spontaneous activity was measured every 24 hours post inoculation for ten days, and recordings were compared to baseline recording obtained before infection. As measures for cell viability, the variables (A) covered electrodes and (B) active electrodes are displayed. At baseline, the firing rate (C), burst frequency (D) and network burst frequency (E) were compared between designated mock and inoculated groups. (F) Firing rate of neural cultures inoculated with heat-inactivated pH1N1 2009 virus were compared to the firing rate of mock and live pH1N1 2009 virus inoculated cultures from Figure 3B. Data from heat-inactivation is from one independent experiment with 5 biological replicates. Other variables for spontaneous activity that were displayed: (G) mean inter-spike-interval within bursts, (H) inter-burst-interval coefficient of variation, (I) number of spikes per burst and (J) mean inter-spike-interval within bursts. Data is depicted as mean ± SEM and are derived from at least four independent experiments, with six technical replicates per experiment (Mock_n_ = 48; pH1N1 2009_n_ = 48; H1N1 2019_n_ = 24; H3N2 2019_n_ = 24, unless datapoints were excluded based on exclusion criteria see material and methods). Statistics was tested with a two-way ANOVA test, and corrected for multiple hypothesis testing using the Benjamini-Hochberg method (*q<Q with Q<0.05, individual p-values in Table S3). Asterisks indicate a positive discovery (*), and nd indicates not a discovery.

**
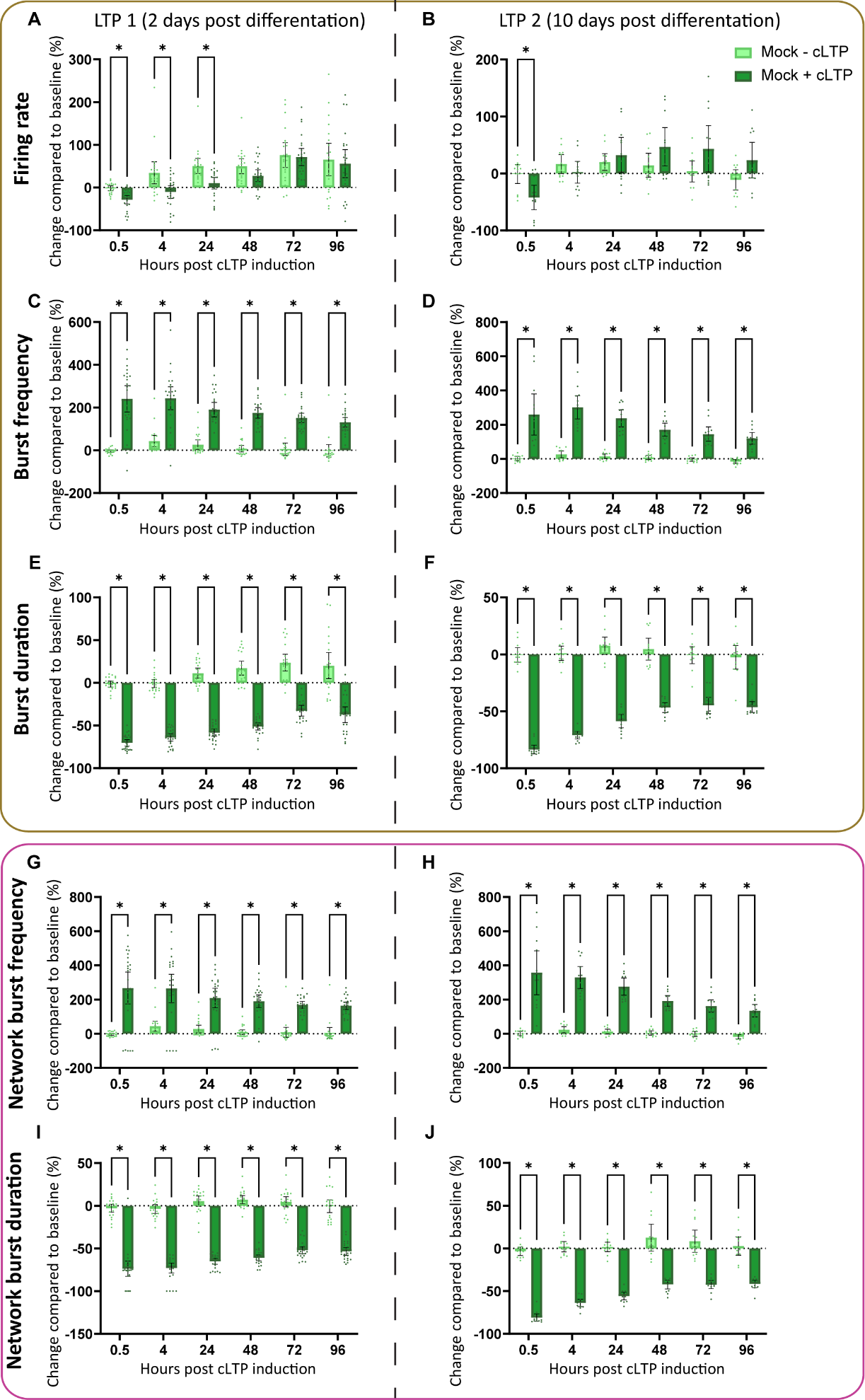
**

**Figure S5. Chemical long-term potentiation is successful induced in neural co-cultures at DIV 23 and 31.** Neural co-cultures were treated with forskolin and rolipram to induce chemical long-term potentiation (cLTP) at 2- or 10 days post differentiation (+cLTP), or treated with DMSO (solvent) as control (-cLTP). Neural activity was measured 0.5-, 4-, 24-, 48-, 72- and 96 hours post cLTP induction. The following variables were displayed: (AB) Firing rate, (CD) Burst frequency, (EF) Burst duration, (GH) Network burst frequency, (IJ) Network burst duration. Data is depicted as mean ± SEM and are derived from at least four independent experiments, with six technical replicates per experiment (n_-cLTP-LTP1_ = 24; n_+cLTP-LTP1_ = 24; n_-cLTP-LTP2_ = 12; n_+cLTP-LTP2_ = 12, unless datapoints were excluded based on exclusion criteria see material and methods). Statistics was tested with a two-way ANOVA test, and corrected for multiple hypothesis testing using the Benjamini-Hochberg method (*q<Q with Q<0.05, individual p-values in Table S6). Asterisks indicate a positive discovery (*).

**Table S1. Individual p-values pixel-based cleaved caspase-3 quantification**

|  | Mock vs pH1N1 2009 | Mock vs H1N1 2019 | Mock vs H3N2 2019 |
| --- | --- | --- | --- |
| Day 3 | 0.0061 (**) | 0.0570 | 0.1057 |
| Day 5 | 0.5538 | 0.0025 (**) | 0.1272 |
| Day 10 | 0.0804 | 0.9275 | 0.3247 |

**Table S2. Individual p-values innate immune responses Figure S3**

| **IFN-α2** | **24 hpi** | **72 hpi** |
| --- | --- | --- |
| Mock vs Poly I:C | >0,9999 | >0,9999 |
| Mock vs pH1N1 2009 | >0,9999 | >0,9999 |
| Mock vs H1N1 2019 | 0.1809 | >0,9999 |
| Mock vs H3N2 2019 | >0,9999 | >0,9999 |
| **IFN-β** | **24 hpi** | **72 hpi** |
| Mock vs Poly I:C | 0.9939 | 0.9584 |
| Mock vs pH1N1 2009 | 0.0818 | 0.9584 |
| Mock vs H1N1 2019 | 0.9944 | 0.1824 |
| Mock vs H3N2 2019 | 0.9944 | 0.9584 |
| **IFN-γ** | **24 hpi** | **72 hpi** |
| Mock vs Poly I:C | <0,0001 (****) | <0,0001 (****) |
| Mock vs pH1N1 2009 | >0,9999 | >0,9999 |
| Mock vs H1N1 2019 | >0,9999 | >0,9999 |
| Mock vs H3N2 2019 | >0,9999 | >0,9999 |
| **IFN-λ1** | **24 hpi** | **72 hpi** |
| Mock vs Poly I:C | 0.1499 | 0.0024 (**) |
| Mock vs pH1N1 2009 | 0.9971 | >0,9999 |
| Mock vs H1N1 2019 | >0,9999 | >0,9999 |
| Mock vs H3N2 2019 | >0,9999 | >0,9999 |
| **IFN-1β** | **24 hpi** | **72 hpi** |
| Mock vs Poly I:C | 0.5809 | 0.1550 |
| Mock vs pH1N1 2009 | >0,9999 | >0,9999 |
| Mock vs H1N1 2019 | >0,9999 | >0,9999 |
| Mock vs H3N2 2019 | >0,9999 | >0,9999 |
| **IL-6** | **24 hpi** | **72 hpi** |
| Mock vs Poly I:C | >0,9999 | >0,9999 |
| Mock vs pH1N1 2009 | >0,9999 | >0,9999 |
| Mock vs H1N1 2019 | 0.1978 | >0,9999 |
| Mock vs H3N2 2019 | >0,9999 | >0,9999 |
| **IL-8** | **24 hpi** | **72 hpi** |
| Mock vs Poly I:C | 0.0031 (**) | 0.0006 (***) |
| Mock vs pH1N1 2009 | >0,9999 | >0,9999 |
| Mock vs H1N1 2019 | >0,9999 | >0,9999 |
| Mock vs H3N2 2019 | 0.5448 | >0,9999 |
| **IL-12p70** | **24 hpi** | **72 hpi** |
| Mock vs Poly I:C | >0,9999 | >0,9999 |
| Mock vs pH1N1 2009 | >0,9999 | >0,9999 |
| Mock vs H1N1 2019 | >0,9999 | >0,9999 |
| Mock vs H3N2 2019 | 0.1488 | >0,9999 |
| **TNF-α** | **24 hpi** | **72 hpi** |
| Mock vs Poly I:C | 0.0039 (**) | 0.0003 (***) |
| Mock vs pH1N1 2009 | >0,9999 | >0,9999 |
| Mock vs H1N1 2019 | >0,9999 | 0.3683 |
| Mock vs H3N2 2019 | >0,9999 | >0,9999 |

**Table S3. Individual p-values spontaneous activity Figure S4**

| **Number of covered electrodes (S3A)** | **Mock vs pH1N1 2009** | **Mock vs H1N1 2019** | **Mock vs H3N2 2019** |
| --- | --- | --- | --- |
| **Baseline** | 0.4236 | 0.1979 | 0.1145 |
| **Day 1** | 0.5296 | 0.2893 | 0.0690 |
| **Day 2** | 0.2374 | 0.0168 (*) | 0.0057 (**) |
| **Day 3** | 0.6092 | 0.0882 | 0.0137 (*) |
| **Day 4** | 0.4741 | 0.1313 | 0.0042 (**) |
| **Day 5** | 0.4233 | 0.0222 (*) | 0.0041 (**) |
| **Day 6** | 0.6707 | 0.2247 | 0.0063 (**) |
| **Day 7** | 0.4423 | 0.0061 (**) | 0.0510 |
| **Day 8** | 0.0628 | 0.0007 (***) | 0.0146 (*) |
| **Day 9** | 0.2985 | 0.0056 (**) | 0.0248 (*) |
| **Day 10** | 0.3887 | 0.0069 (**) | 0.0167 (*) |
| **Number of active electrodes (S3B)** | **Mock vs pH1N1 2009** | **Mock vs H1N1 2019** | **Mock vs H3N2 2019** |
| **Baseline** | 0.2915 | 0.2593 | 0.5982 |
| **Day 1** | 0.1626 | 0.2837 | 0.7062 |
| **Day 2** | 0.3534 | 0.0406 (*) | 0.9893 |
| **Day 3** | 0.0885 | 0.0259 (*) | 0.9228 |
| **Day 4** | 0.0459 (*) | 0.0047 (**) | 0.6168 |
| **Day 5** | 0.004 (**) | 0.0071 (**) | 0.4780 |
| **Day 6** | 0.0022 (**) | 0.0097 (**) | 0.9116 |
| **Day 7** | 0.0004 (***) | 0.0015 (**) | 0.0337 (*) |
| **Day 8** | 0.001 (***) | 0.0211 (*) | 0.1778 |
| **Day 9** | 0.0861 | 0.2561 | 0.2123 |
| **Day 10** | 0.0018 (**) | 0.0571 | 0.0261 (*) |
| **Firing rate (S3C)** | **Mock vs pH1N1 2009** | **Mock vs H1N1 2019** | **Mock vs H3N2 2019** |
| **Baseline** | 0.7371 | 0.2261 | 0.5895 |
| **Burst frequency (S3D)** | **Mock vs pH1N1 2009** | **Mock vs H1N1 2019** | **Mock vs H3N2 2019** |
| **Baseline** | 0.9931 | 0.0867 | 0.0722 |
| **Network burst frequency (S3E)** | **Mock vs pH1N1 2009** | **Mock vs H1N1 2019** | **Mock vs H3N2 2019** |
| **Baseline** | 0.4923 | 0.6003 | 0.5879 |
| **Firing rate (S3F)** | **Heat-inactivated vs mock (Fig. 3B)** | **Heat-inactivated vs pH1N1 2009 (Fig. 3B)** | **Mock (Fig. 3B) vs pH1N1 2009 (Fig. 3B)** |
| **Day 1** | 0.8020 | 0.0223 (*) | <0.0001 (****) |
| **Day 2** | 0.4843 | <0.0001 (****) | <0.0001 (****) |
| **ISI coefficient of variation (S3G)** | **Mock vs pH1N1 2009** | **Mock vs H1N1 2019** | **Mock vs H3N2 2019** |
| **Day 1** | <0.0001 (****) | 0.0772 | 0.01 (**) |
| **Day 2** | <0.0001 (****) | 0.2979 | 0.2693 |
| **Day 3** | <0.0001 (****) | 0.2143 | 0.7215 |
| **Day 4** | 0.0006 (***) | 0.0087 (**) | 0.1046 |
| **Day 5** | 0.0012 (**) | 0.0119 (*) | 0.0487 (*) |
| **Day 6** | <0.0001 (****) | 0.0202 (*) | 0.0630 |
| **Day 7** | <0.0001 (****) | 0.0820 | 0.0432 (*) |
| **Day 8** | 0.0195 (*) | 0.0081 (**) | 0.0054 (**) |
| **Day 9** | 0.0006 (***) | 0.0605 | 0.0469 (*) |
| **Day 10** | 0.0431 (*) | 0.0077 (**) | 0.0068 (**) |
| **IBI coefficient of variation (S3H)** | **Mock vs pH1N1 2009** | **Mock vs H1N1 2019** | **Mock vs H3N2 2019** |
| **Day 1** | 0.6204 | 0.0034 (**) | 0.0064 (**) |
| **Day 2** | 0.6191 | 0.0105 (*) | 0.0088 (**) |
| **Day 3** | 0.7073 | 0.0331 (*) | 0.0044 (**) |
| **Day 4** | 0.2807 | 0.0123 (*) | 0.0009 (***) |
| **Day 5** | 0.5522 | 0.0009 (***) | 0.0825 |
| **Day 6** | 0.9436 | 0.0995 | 0.8209 |
| **Day 7** | 0.1428 | 0.0129 (*) | 0.0379 (*) |
| **Day 8** | 0.4495 | 0.0288 (*) | 0.0817 |
| **Day 9** | 0.1889 | 0.0576 | 0.9970 |
| **Day 10** | 0.6392 | 0.0051 (**) | 0.0086 (**) |
| **Number of spikes per burst (S3I)** | **Mock vs pH1N1 2009** | **Mock vs H1N1 2019** | **Mock vs H3N2 2019** |
| **Day 1** | 0.0025 (**) | 0.0264 (*) | 0.0152 (*) |
| **Day 2** | <0.0001 (****) | 0.6780 | 0.0121 (*) |
| **Day 3** | 0.0003 (***) | 0.5259 | 0.2034 |
| **Day 4** | 0.034 (*) | 0.1548 | 0.8966 |
| **Day 5** | 0.0052 (**) | 0.1943 | 0.9683 |
| **Day 6** | 0.0061 (**) | 0.1805 | 0.9696 |
| **Day 7** | 0.0172 (*) | 0.0991 | 0.1961 |
| **Day 8** | 0.0145 (*) | 0.0319 (*) | 0.1328 |
| **Day 9** | 0.0153 (*) | 0.0814 | 0.3489 |
| **Day 10** | 0.0211 (*) | 0.0397 (*) | 0.0966 |
| **Mean ISI within bursts (S3J)** | **Mock vs pH1N1 2009** | **Mock vs H1N1 2019** | **Mock vs H3N2 2019** |
| **Day 1** | 0.7015 | 0.4148 | <0.0001 (****) |
| **Day 2** | 0.0121 (*) | 0.3389 | 0.0759 |
| **Day 3** | 0.1247 | 0.353 | 0.0036 (**) |
| **Day 4** | 0.0426 (*) | 0.2354 | 0.0522 |
| **Day 5** | 0.6107 | 0.6335 | 0.0454 (*) |
| **Day 6** | 0.4503 | 0.9815 | 0.4719 |
| **Day 7** | 0.1287 | 0.8816 | 0.5561 |
| **Day 8** | 0.4994 | 0.6413 | 0.6508 |
| **Day 9** | 0.8649 | 0.7869 | 0.259 |
| **Day 10** | 0.1576 | 0.2821 | 0.5095 |

**Table S4. Individual p-values spontaneous activity Figure 3**

| **Firing rate (3B)** | **Mock vs pH1N1 2009** | **Mock vs H1N1 2019** | **Mock vs H3N2 2019** |
| --- | --- | --- | --- |
| **Day 1** | <0.0001 (****) | 0.1674 | 0.0264 (*) |
| **Day 2** | <0.0001 (****) | 0.0005 (***) | 0.3936 |
| **Day 3** | <0.0001 (****) | 0.0637 | 0.8536 |
| **Day 4** | 0.0001 (***) | <0.0001 | 0.0019 (**) |
| **Day 5** | 0.0045 (**) | 0.0021 (**) | 0.0128 (*) |
| **Day 6** | <0.0001 (****) | 0.0079 (**) | 0.0156 (*) |
| **Day 7** | <0.0001 (****) | 0.0037 (**) | 0.007 (**) |
| **Day 8** | 0.1648 | 0.0008 (***) | 0.0029 (**) |
| **Day 9** | 0.0003 (***) | 0.0061 (**) | 0.0123 (*) |
| **Day 10** | 0.1313 | 0.0056 (**) | 0.0054 (**) |
| **Burst frequency (3C)** | **Mock vs pH1N1 2009** | **Mock vs H1N1 2019** | **Mock vs H3N2 2019** |
| **Day 1** | 0.0003 (***) | 0.8130 | 0.1296 |
| **Day 2** | 0.0052 (**) | 0.2427 | 0.0123 (*) |
| **Day 3** | 0.0089 (**) | 0.6147 | 0.069 |
| **Day 4** | 0.0529 | 0.0072 (**) | 0.0022 (**) |
| **Day 5** | 0.0113 (*) | 0.4712 | 0.0145 (*) |
| **Day 6** | 0.0121 (*) | 0.9754 | 0.022 (*) |
| **Day 7** | 0.1256 | 0.9072 | 0.0454 (*) |
| **Day 8** | 0.0312 (*) | 0.5165 | 0.0108 (*) |
| **Day 9** | 0.0298 (*) | 0.7576 | 0.0484 (*) |
| **Day 10** | 0.0326 (*) | 0.4391 | 0.0131 (*) |
| **Burst duration (3D)** | **Mock vs pH1N1 2009** | **Mock vs H1N1 2019** | **Mock vs H3N2 2019** |
| **Day 1** | 0.0001 (***) | 0.5251 | 0.3607 |
| **Day 2** | <0.0001 (****) | 0.0711 | 0.9424 |
| **Day 3** | <0.0001 (****) | 0.0034 (**) | 0.1381 |
| **Day 4** | <0.0001 (****) | 0.0002 (***) | 0.0042 (**) |
| **Day 5** | <0.0001 (****) | 0.0002 (***) | 0.0138 (*) |
| **Day 6** | <0.0001 (****) | 0.0066 (**) | 0.1023 |
| **Day 7** | 0.0092 (**) | 0.0013 (**) | 0.0023 (**) |
| **Day 8** | 0.0006 (***) | 0.0001 (***) | 0.0012 (**) |
| **Day 9** | 0.006 (**) | 0.0009 (***) | 0.0121 (*) |
| **Day 10** | 0.0009 (***) | 0.0001 (***) | 0.0003 (***) |

**Table S5. Individual p-values network activity Figure 4**

| **Network burst frequency (4B)** | **Mock vs pH1N1 2009** | **Mock vs H1N1 2019** | **Mock vs H3N2 2019** |
| --- | --- | --- | --- |
| **Day 1** | 0.0004 (***) | 0.0558 | 0.5891 |
| **Day 2** | 0.0041 (**) | 0.0122 (*) | 0.4599 |
| **Day 3** | 0.02 (*) | 0.0793 | 0.8854 |
| **Day 4** | 0.0685 | 0.0034 (**) | 0.3523 |
| **Day 5** | 0.0345 (*) | 0.0197 (*) | 0.3650 |
| **Day 6** | 0.0304 (*) | 0.0321 (*) | 0.5672 |
| **Day 7** | 0.1653 | 0.049 (*) | 0.4591 |
| **Day 8** | 0.1195 | 0.0157 (*) | 0.3270 |
| **Day 9** | 0.0486 (*) | 0.0535 | 0.4435 |
| **Day 10** | 0.1139 | 0.0228 (*) | 0.1921 |
| **Network burst duration (4C)** | **Mock vs pH1N1 2009** | **Mock vs H1N1 2019** | **Mock vs H3N2 2019** |
| **Day 1** | 0.0003 (***) | 0.1719 | 0.0521 |
| **Day 2** | <0.0001 (****) | 0.1746 | 0.1366 |
| **Day 3** | <0.0001 (****) | 0.1225 | 0.8585 |
| **Day 4** | 0.0683 | 0.0048 (**) | 0.2274 |
| **Day 5** | 0.0124 (*) | 0.0927 | 0.5188 |
| **Day 6** | 0.0192 (*) | 0.1154 | 0.4991 |
| **Day 7** | 0.0144 (*) | 0.0582 | 0.1055 |
| **Day 8** | 0.0171 (*) | 0.0166 (*) | 0.0426 (*) |
| **Day 9** | 0.0062 (**) | 0.04 (*) | 0.0851 |
| **Day 10** | 0.0153 (*) | 0.0196 (*) | 0.0296 (*) |
| **Number of spikes per network burst (4D)** | **Mock vs pH1N1 2009** | **Mock vs H1N1 2019** | **Mock vs H3N2 2019** |
| **Day 1** | 0.0016 (**) | 0.0001 (***) | 0.0005 (***) |
| **Day 2** | 0.0062 (**) | <0.0001 (****) | 0.0334 (*) |
| **Day 3** | 0.0015 (**) | <0.0001 (****) | 0.002 (**) |
| **Day 4** | 0.0036 (**) | <0.0001 (****) | 0.0019 (**) |
| **Day 5** | 0.0005 (***) | 0.0016 (**) | 0.0448 (*) |
| **Day 6** | 0.0451 (*) | 0.1410 | 0.2210 |
| **Day 7** | 0.4326 | 0.0095 (**) | 0.0024 (**) |
| **Day 8** | 0.2313 | 0.0129 (*) | 0.0028 (**) |
| **Day 9** | 0.4194 | 0.1533 | 0.0234 (*) |
| **Day 10** | 0.1108 | 0.0996 | 0.0145 (*) |

**Table S6. Individual p-values cLTP induction in mock neural co-cultures (Figure S5)**

|  | **Time post cLTP induction** | | | | | |
| --- | --- | --- | --- | --- | --- | --- |
| Mock -cLTP vs Mock +cLTP | **0.5** | **4** | **24** | **48** | **72** | **96** |
| **Firing rate DIV 23 (S4A)** | <0.0001 (****) | 0.0036 (**) | 0.0005 (***) | 0.0463 (*) | 0.7899 | 0.6964 |
| **Firing rate DIV 31 (S4B)** | 0.0031 (**) | 0.2236 | 0.4500 | 0.0899 | 0.0698 | 0.0491 (*) |
| **Burst frequency DIV 23 (S4C)** | <0.0001 (****) | <0.0001 (****) | <0.0001 (****) | <0.0001 (****) | <0.0001 (****) | <0.0001 (****) |
| **Burst frequency DIV 31 (S4D)** | 0.0006 (***) | <0.0001 (****) | <0.0001 (****) | <0.0001 (****) | <0.0001 (****) | <0.0001 (****) |
| **Burst duration DIV 23 (S4E)** | <0.0001 (****) | <0.0001 (****) | <0.0001 (****) | <0.0001 (****) | <0.0001 (****) | <0.0001 (****) |
| **Burst duration DIV 31 (S4F)** | <0.0001 (****) | <0.0001 (****) | <0.0001 (****) | <0.0001 (****) | <0.0001 (****) | <0.0001 (****) |
| **Network burst frequency DIV 23 (S4G)** | <0.0001 (****) | <0.0001 (****) | <0.0001 (****) | <0.0001 (****) | <0.0001 (****) | <0.0001 (****) |
| **Network burst frequency DIV 31 (S4H)** | <0.0001 (****) | <0.0001 (****) | <0.0001 (****) | <0.0001 (****) | <0.0001 (****) | <0.0001 (****) |
| **Network burst duration DIV 23 (S4I)** | <0.0001 (****) | <0.0001 (****) | <0.0001 (****) | <0.0001 (****) | <0.0001 (****) | <0.0001 (****) |
| **Network burst duration DIV 31 (S4J)** | <0.0001 (****) | <0.0001 (****) | <0.0001 (****) | <0.0001 (****) | <0.0001 (****) | <0.0001 (****) |

**Table S7. Individual p-values cLTP induction (Figure 6)**

|  | **Time post cLTP induction** | | | | | |
| --- | --- | --- | --- | --- | --- | --- |
| Mock vs pH1N1 2009 | **0.5** | **4** | **24** | **48** | **72** | **96** |
| **Firing rate 2 dpi (6A)** | 0.9332 | 0.0208 (*) | 0.0114 (*) | 0.0306 (*) | 0.0042 (**) | 0.035 (*) |
| **Firing rate 10 dpi (6B)** | <0.0001 (****) | 0.0266 (*) | 0.0207 (*) | <0.0001 (****) | 0.0001 (***) | 0.007 (**) |
| **Burst frequency 2 dpi (6C)** | 0.2812 | 0.8662 | 0.8795 | 0.4755 | 0.6646 | 0.4887 |
| **Burst frequency 10 dpi (6D)** | 0.5775 | 0.3650 | 0.4953 | 0.5307 | 0.6717 | 0.7290 |
| **Burst duration 2 dpi (6E)** | 0.7187 | 0.6531 | 0.7831 | 0.4899 | 0.8226 | 0.3596 |
| **Burst duration 10 dpi (6F)** | 0.1520 | 0.0084 (**) | 0.0435 (*) | 0.6761 | 0.6679 | 0.1079 |
| **Network burst frequency 2 dpi (6G)** | 0.2091 | 0.5029 | 0.4633 | 0.3072 | 0.3877 | 0.3055 |
| **Network burst frequency 10 dpi (6H)** | 0.0124 (*) | 0.005 (**) | 0.0136 (*) | 0.0138 (*) | 0.0228 (*) | 0.0242 (*) |
| **Network burst duration 2 dpi (6I)** | 0.8561 | 0.8236 | 0.1142 | 0.7781 | 0.9312 | 0.3413 |
| **Network burst duration 10 dpi (6J)** | 0.0107 (*) | 0.6080 | 0.0434 (*) | 0.0122 (*) | 0.003 (**) | <0.0001 (****) |
